# A mathematical model of biomedical interventions for HIV prevention among men who have sex with men in China

**DOI:** 10.1186/s12879-018-3516-8

**Published:** 2018-11-28

**Authors:** Jinghua Li, Liping Peng, Stuart Gilmour, Jing Gu, Yuhua Ruan, Huachun Zou, Chun Hao, Yuantao Hao, Joseph Tak-fai Lau

**Affiliations:** 1 Department of Health Policy and Management & Sun Yat-sen Global Health Institute, School of Public Health and Institute of State Governance, Sun Yat-sen University, Guangzhou, China; 2 Department of Health Policy and Management, School of Public Health, Sun Yat-sen University, Guangzhou, China; 3 Graduate School of Public Health, St. Luke’s International University, Tokyo, Japan; 4 Department of Medical Statistics and Epidemiology & Sun Yat-sen Global Health Institute, School of Public Health and Institute of State Governance, Sun Yat-sen University, Guangzhou, China; 5 Division of Virology and Immunology, National Center for AIDS/STD Control and Prevention (NCAIDS), Chinese Center for Disease Control and Prevention, Beijing, China; 6 School of Public Health (Shenzhen), Sun Yat-sen University, Shenzhen, China; 7 Kirby Institute, University of New South Wales, Sydney, Australia; 8 The School of Public Health and Primary Care, The Chinese University of Hong Kong, Hong Kong, China

**Keywords:** HIV, Pre-exposure prophylaxis, Biomedical intervention, Mathematical model, China, Men who have sex with men

## Abstract

**Background:**

The new HIV treatment guidelines in China recommend antiretroviral therapy (ART) for all people living with HIV, but significant gaps in implementation still exist. Pre-exposure prophylaxis (PrEP) can effectively reduce the risk of HIV transmission among men who have sex with men (MSM). This study assessed the epidemiological impact and cost effectiveness of PrEP, enhanced biomedical interventions and their combination among MSM in China.

**Methods:**

A deterministic mathematical model was developed and projected over 20 years to assess the impact of the PrEP, biomedical interventions and their combinations. Incidence and prevalence of HIV were measured, and cost-effectiveness was assessed using incremental cost (international dollars, Int.$) per quality-adjusted life year (QALY) gained.

**Results:**

A total of 0.78 million new HIV infections were estimated to occur over the next 20 years if no additional interventions are implemented among MSM. The PrEP-only strategy covering 25–75% of HIV-negative high-risk MSM can prevent 0.09–0.20 million (12.1–25.7%) new infections, at a cost of 17,277–18,452 Int.$/QALY. The optimal cost-effectiveness path is from test-and-treat to the combination strategy of test-and-treat and PrEP. Some strategies could almost eliminate new HIV infections over the next 20 years.

**Conclusions:**

PrEP, test-and-treat, and their combinations among MSM are effective and cost-effective relative to current policy. PrEP is an important and cost-effective addition to current policy in China.

**Electronic supplementary material:**

The online version of this article (10.1186/s12879-018-3516-8) contains supplementary material, which is available to authorized users.

## Background

HIV/AIDS continues to be a serious public health problem in China, especially among key risk populations. It is estimated that 780,000 people were living with HIV (PLWH) in China in 2011, of whom 32.5% were men who have sex with men (MSM) [1]. HIV prevalence among MSM in China increased sharply from 5.5% in 2009 to 8.0% in 2015 [2], and rapid upward trends in HIV incidence were also found among this population, increasing from 0.39 (0.21–0.56) per 100 person-years in 2000 to 0.98 (0.70–1.25) in 2010 [3]. The percentage of reported cases attributed to MSM also increased from 2.5% in 2006 to 25.8% in 2014 [4] and MSM are now the fastest growing group of PLWH in China.

The Chinese government has increased policy attention and scaled up responses to the HIV/AIDS epidemic [5], including the recent revision of national treatment criteria, which now recommend initiation of ART for all PLWH, regardless of World Health Organization (WHO) clinical stage and at any CD4 cell count [6]. However, in real-world settings, significant gaps still exist [7] to achieve the Joint United Nations Programme on HIV/AIDS (UNAIDS) 90–90-90 targets by 2020. By the end of 2015, China had not reached the 90–90-90 targets, with only 68% of PLWH diagnosed, 67% of diagnosed PLWH in treatment and 91% of people in treatment attaining viral load targets [7]. For example, the lifetime- and annual- HIV testing rates among MSM in China are only 47 and 38% respectively [8]. It is estimated that 61.6–87% of all HIV cases among MSM remain undiagnosed [9, 10]. Around 56% [11] of diagnosed HIV-positive MSM received ART. Because of these gaps in intervention effectiveness, new strategies need to be considered, and recently attention has turned to pre-exposure prophylaxis (PrEP) – the use of tenofovir and emtricitabine among HIV-negative individuals. Large scale randomized controlled trials have consistently shown that both daily PrEP [12] and on-demand PrEP [13] can effectively reduce the risk of HIV transmission among high-risk MSM by 44–92% depending on adherence. New WHO guidelines [14] recommend offering PrEP to people at substantial risk of HIV infection, but this option has not yet been made available in China.

It is important to assess the potential impact of PrEP strategies and their combination with other biomedical interventions in the Chinese context. In this study, we used mathematical modeling to investigate the epidemiological impact and cost-effectiveness of various biomedical interventions and their combinations on HIV transmission over the next 20 years in China. This study assesses the benefits both of full implementation of current policies and the timely introduction of novel policies, and makes recommendations for future HIV policy responses in China.

## Methods

**Fig. 1:**
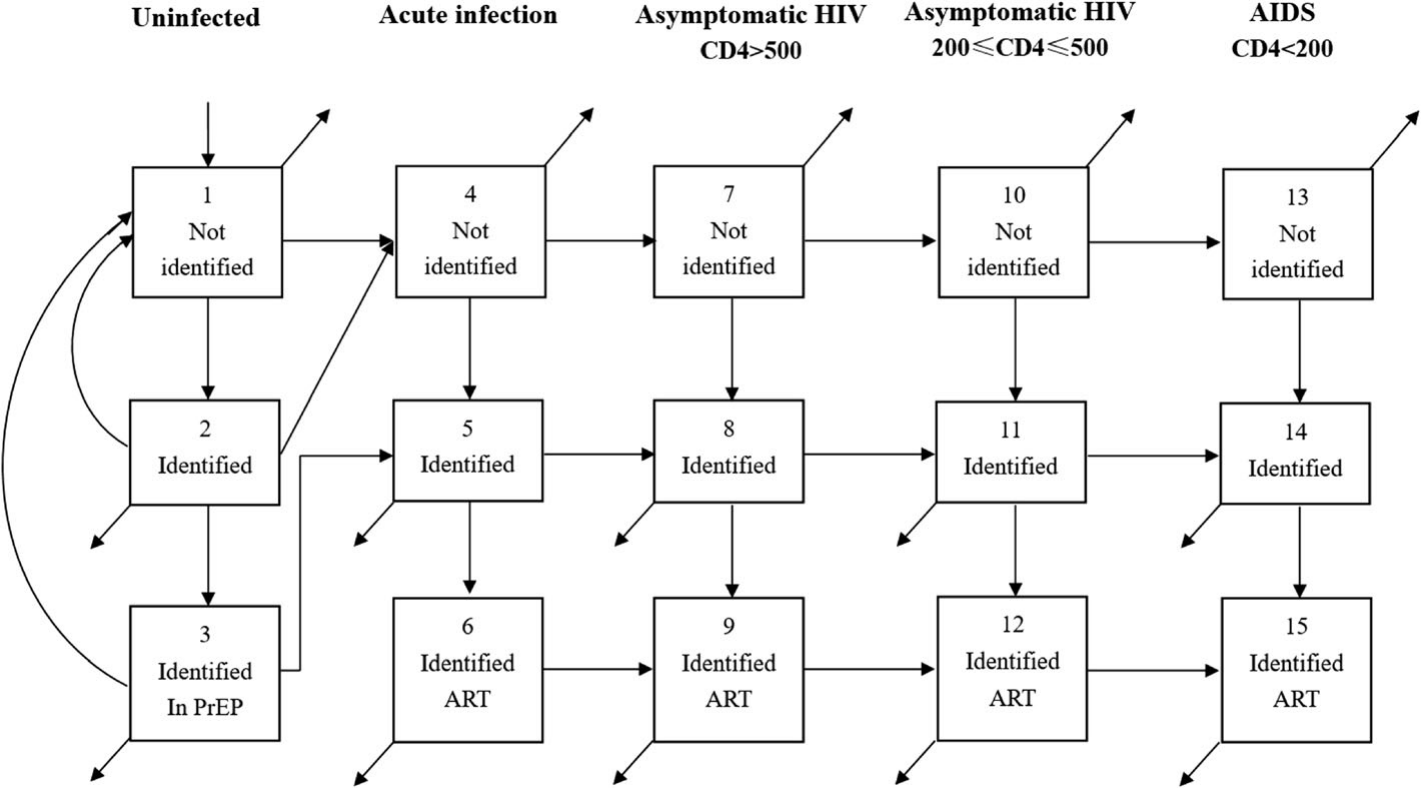
HIV transmission compartmental model structure

We developed a deterministic compartmental mathematical model that divides the population at risk into five groups based on HIV serostatus, disease stage and CD4 count, and further divides the population into three groups based on serostatus and treatment state. This model builds on previous models in China [15], the USA [16] and sub-Saharan Africa [17], structures disease stages to reflect WHO treatment recommendations [18], and incorporates a stage for acute infection. A separate “treatment” compartment for HIV-negative subjects allows the incorporation of PrEP into the model. The compartmental structure of the model is shown in Fig. 1, with mathematical symbols removed for simplicity. Arrows between boxes indicate population flows, with arrows not terminating in a compartment indicating mortality. Flow into and out of each compartment is described by an ordinary differential equation (ODE), with each arrow representing a single flow process contributing to a single ODE, and described by a single rate parameter. The rate of flow from compartments 1–3 (uninfected) to compartments 4 or 5 (acute infection) is determined dynamically by a force of infection term composed from the infectiousness of any HIV-positive sexual contact, rates of sexual partnership, and probability that any single partnership is infectious. The full system of ODEs and detailed formulae for the forces of infection are given in Additional file 1 Appendix Section 2.2.

The compartment structure shown in Fig. 1 was replicated for high-risk and low-risk MSM, and the total population of MSM was divided between high-risk and low-risk at a ratio of 1:4 (20% of the population was high-risk) in a total population of 3,625,000. This division was based on annual sexual partnerships, with high-risk men having 15 per year and low-risk men having 2.6, for a balanced average of 5 partnerships per year [19]. All population estimates and parameters were drawn from published literature and are summarized in supplementary Table S2. In this model without ART, the asymptomatic disease stage (CD4 > 200) will last for about 8 years [20]. International randomized controlled trials (RCTs) [21, 22] demonstrated a 93–100% reduction in HIV transmission risk for ART. ART effectiveness was assumed to be 90% to reflect the rate of viral suppression among those on ART in China [7]. PrEP effectiveness was assumed to be 60% to reflect the adherence rates from demonstration PrEP studies in China [23, 24]. Some assumptions were also made about reductions in sexual partnerships at advanced disease stages and upon identification of HIV-positive serostatus (Table S2).

The system of ODEs developed to describe Fig. 1 was solved numerically in Matlab 2016 using a difference equation method with a time step of one month. The model was projected forward 20 years to 2037 and data on HIV incidence, HIV prevalence and HIV mortality extracted. The model was calibrated against HIV prevalence data from 2005 to 2015, using a simple search procedure to identify 400 parameter sets based on the deviance criterion [25]. The 400 parameter sets that closest calibrated to observed data were used as uncertainty ranges, and epidemiological outcomes were estimated as the mean of the 400 uncertainty runs, weighted by the inverse deviance of each run. Formulae for epidemiological outcomes and heuristics for model simulation and calibration are described in detail in Additional file 1 Appendix sections 2.4–2.6.

### Modeled policy scenarios

We projected a base-case model that assumed current Chinese HIV treatment guidelines were followed for the next 20 years with no change in testing uptake and treatment entry rates. Against this base case we modeled several alternative scenarios and all of their combinations:

1. Scenario 1: Test-and-treat strategy that is fully compliant with the WHO 90–90-90 recommendations, effectively representing the current HIV treatment guidelines implemented at much greater effectiveness than the current standard. This scenario assumed annual testing rates of 90% for all MSM, with an ART utilization rate of 90% for all diagnosed PLWH, and 90% of all PLWH receiving ART achieving viral suppression (i.e., 90% ART effectiveness).
2. Scenario 2–4: PrEP for high-risk MSM with coverage of 25, 50% or 75% of the entire population of identified HIV-negative high-risk MSM.
3. Scenario 5–7: PrEP for high-risk MSM (with the same three levels of PrEP coverage) combined with expanded annual VCT of 90% MSM.
4. Scenario 8–10: PrEP for high-risk MSM (with the same three levels of PrEP coverage) combined with the test-and-treat strategy.

PrEP was modeled separately with and without widespread VCT because current levels of HIV testing amongst MSM are low, and PrEP implemented against this low backdrop of VCT may have limited effectiveness. Test-and-treat to the 90–90-90 standard was modeled as a separate policy scenario to the base case to reflect low current testing and treatment entry rates, especially in rural areas with limited ability to finance the required interventions [7]. We defined elimination of HIV as HIV incidence of less than 1 per 1000 person-years [17].

### Health economic analysis

We estimated the cost of all policy scenarios and conducted a full health economic analysis. Policy scenario costs were estimated on the basis of known costs of testing, ART treatment and average cost of treating AIDS and associated opportunistic infections. PrEP costs were assumed on the basis of the domestic price of Truvada. Costs were only estimated for that proportion of the population assumed to be covered by a given intervention. We compared the estimated cost of each policy with the HIV/AIDS-related healthcare costs averted by reduced infections or slowed disease progression, relative to the base case only. Cost-effectiveness analysis was conducted following standard protocols [26], calculating the average cost-effectiveness ratio (CER) for each policy scenario relative to the base case, and then calculating the incremental cost-effectiveness ratio (ICER) of each scenario relative to the next most cost-effective scenario. All costs were reported in 2017 International Dollars (Int.$), calculated using the International Monetary Fund gross domestic product (GDP) deflator and implied purchasing power parity conversion rates. All cost and health outcomes (e.g., quality-adjusted life year, QALY) were calculated with an annual discount rate of 3%. All cost data and quality of life multipliers were drawn from published literature and are summarized in supplementary Table S2.

### Sensitivity analysis

We conducted two scenario-based sensitivity analyses. We tested the impact of the PrEP and combined scenarios when PrEP adherence was 30, 60% or 90%, to explore the possible effectiveness with various adherence rates. Secondly, we tested the impact of the PrEP and combined scenarios after adjusting for 100% risk compensation, in which all high-risk MSM in the PrEP program completely ceased all forms of safer sex behavior, to test the potential impact on a PrEP program of risk compensation. Full details and results of these sensitivity analyses are given in Additional file 1: Appendix section 3.

## Results

### Baseline HIV epidemic projections

**Fig. 2:**
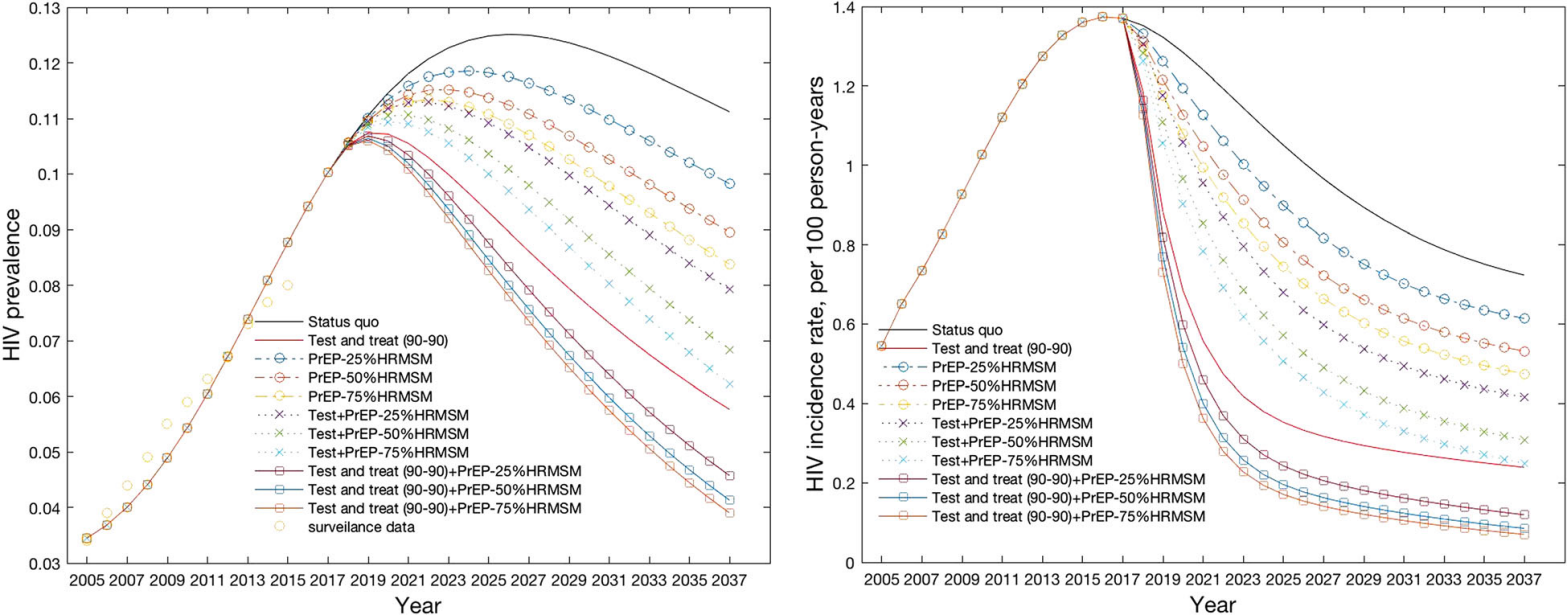
Estimated HIV prevalence and incidence between 2005 and 2037 in the status quo and the ten intervention scenarios. Left) HIV prevalence; Right) HIV incidence

The model estimates of the HIV prevalence and incidence rate among Chinese MSM in the past eleven years showed good fit with the national estimates and surveillance data [2] (Fig. 2), and all selected models were not statistically significantly different from the calibration data on a goodness-of-fit test based on their calibration deviance statistic. Without any additional intervention, HIV prevalence will increase to 11.2% in 2037 and there will be 0.78 million cumulative new infections among MSM over the next 20 years (Table 1). In this scenario the HIV incidence rate will be 0.73 per 100 person-years in 2037. Among all scenarios, only combination scenarios (scenarios 9–10) will achieve HIV elimination: the combination strategy of test-and-treat and PrEP (50% of high-risk MSM), by 2037; and the combination strategy of test-and-treat and PrEP (75% of high-risk MSM), by 2033.

**Table 1:** Benefits, costs and savings in healthcare costs of the individual and combined interventions, 2018~2037

	Total infection over 20 years (2018~2037), million	HIV infections prevented over 20 years, million	HIV infections prevented over 20 years, %	HIV prevalence at 2037, %	HIV incidence rate at 2037, per 100 person-years	Incremental costs, Int.$ (billion)	Incremental QALYs, million	Average CER, Int.$/QALY, relative to base case	Saving in healthcare cost, Int.$ (billion)
Base case	0.78 (0.60–1.00)	–	–	11.2 (9.1–14.2)	0.73 (0.56–0.98)	–	–	–	–
Test and treat (90–90)	0.33 (0.28–0.39)	0.45 (0.28–0.62)	57.9% (46.4–66.5%)	5.8 (4.8–6.8)	0.24 (0.18–0.29)	2.13 (0.61–4.03)	1.22 (0.94–1.53)	1754 (462–3960)	8.86 (5.33–13.92)
PrEP-25% HRMSM	0.68 (0.57–0.87)	0.09 (0.06–0.14)	12.1% (8.7–16.7%)	9.8 (8.2–12.8)	0.61 (0.50–0.80)	3.08 (1.76–5.30)	0.18 (0.12–0.23)	17,277 (12,127–23,711)	1.73 (0.94–2.79)
PrEP-50% HRMSM	0.62 (0.52–0.80)	0.16 (0.10–0.23)	20.2% (15.1–26.9%)	8.9 (7.5–11.7)	0.53 (0.44–0.70)	5.34 (3.07–9.10)	0.30 (0.21–0.38)	17,979 (12,631–24,991)	2.87 (1.60–4.52)
PrEP-75% HRMSM	0.57 (0.49–0.75)	0.20 (0.13–0.29)	25.7% (19.6–33.3%)	8.4 (7.0–11.0)	0.47 (0.40–0.63)	6.98 (4.05–11.80)	0.38 (0.27–0.48)	18,452 (12,972–25,846)	3.64 (2.06–5.64)
Test+PrEP-25% HRMSM	0.53 (0.46–0.68)	0.24 (0.15–0.34)	30.8% (23.9–39.3%)	7.9 (6.8–10.2)	0.42 (0.36–0.53)	6.83 (4.39–10.62)	0.49 (0.37–0.61)	13,835 (9600–18,598)	4.36 (2.46–6.82)
Test+PrEP-50% HRMSM	0.46 (0.39–0.59)	0.32 (0.21–0.44)	41.0% (33.3–49.9%)	6.8 (5.8–8.8)	0.31 (0.26–0.40)	10.61 (6.65–16.76)	0.64 (0.48–0.78)	16,636 (11,595–22,919)	5.77 (3.37–8.86)
Test+PrEP-75% HRMSM	0.41 (0.35–0.53)	0.36 (0.25–0.49)	47.0% (39.3–55.6%)	6.2 (5.2–8.1)	0.25 (0.20–0.33)	13.20 (8.25–20.85)	0.73 (0.56–0.88)	18,110 (12,660–25,289)	6.64 (3.95–10.12)
Test and treat (90–90) + PrEP 25% HRMSM	0.24 (0.21–0.30)	0.53 (0.39–0.68)	68.3% (61.1–75.4%)	4.6 (3.7–5.6)	0.12 (0.08–0.15)	10.11 (7.42–13.49)	1.33 (1.12–1.69)	7574 (5166–10,243)	10.17 (6.30–16.33)
Test and treat (90–90) + PrEP 50% HRMSM	0.21 (0.18–0.26)	0.56 (0.43–0.72)	72.8% (66.6–78.7%)	4.1 (3.4–5.1)	0.09 (0.06–0.11)	14.69 (10.27–20.60)	1.40 (1.19–1.76)	10,485 (7199–14,484)	10.83 (6.82–17.25)
Test and treat (90–90) + PrEP 75% HRMSM	0.19 (0.16–0.24)	0.58 (0.45–0.74)	75.2% (69.7–80.5%)	3.9 (3.2–4.8)	0.07 (0.05–0.09)	17.63 (12.14–25.06)	1.44 (1.23–1.81)	12,218 (8415–16,992)	11.23 (7.13–17.81)

*PrEP* pre-exposure prophylaxis, *QALY* quality-adjusted life years, *CER* cost-effectiveness ratio, *Int.$* international dollars, *HRMSM*, high-risk MSM

^a^Interventions that cost less than per capita gross domestic product (i.e., 15,943 Int.$) per QALYgained are defined as very cost effective [40]

^b^Interventions that cost between one and three times per capita gross domestic product per QALY gained are defined as cost effective [40]

### Impact of biomedical interventions

**Fig. 3:**
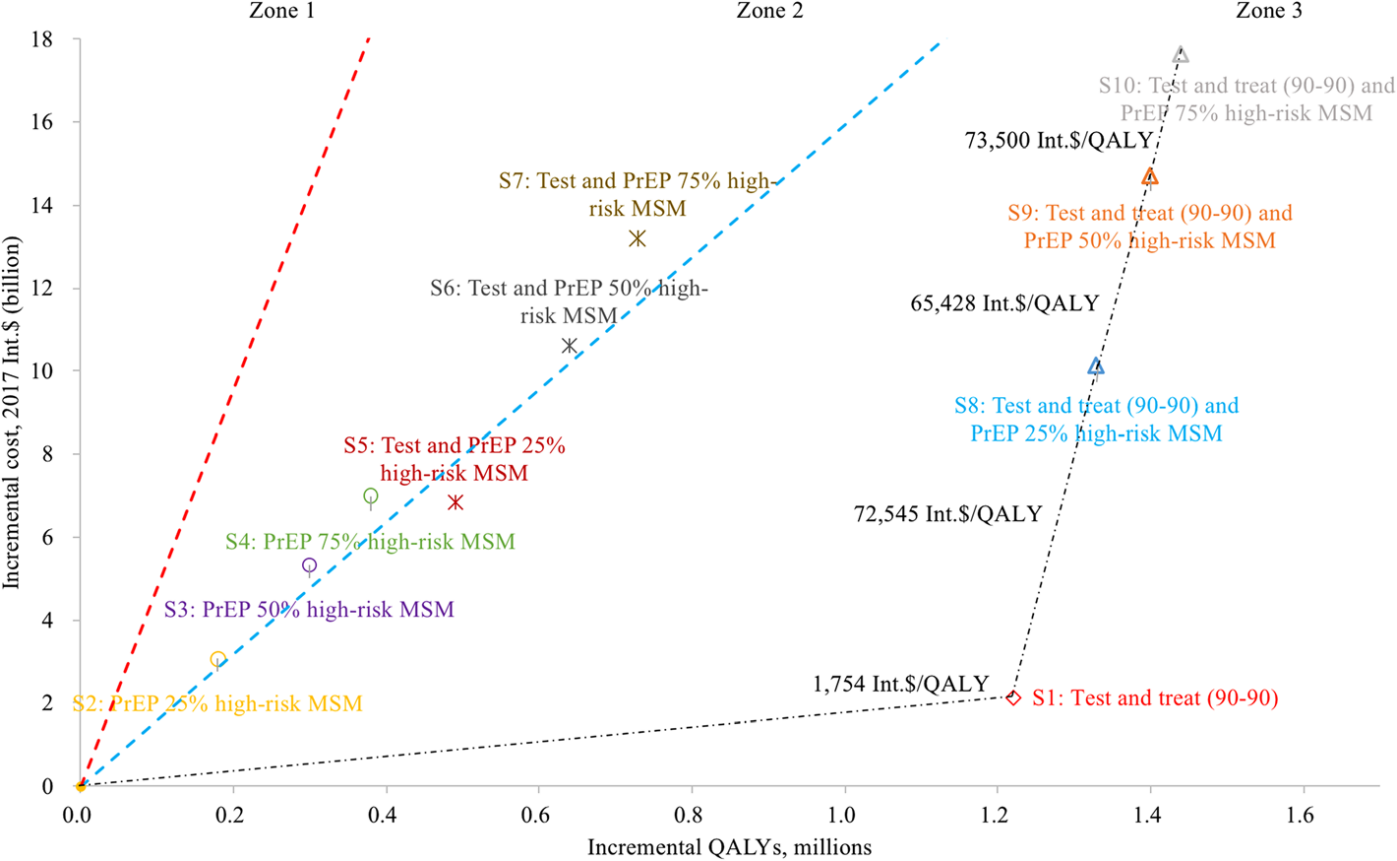
Cost-effectiveness frontier for all strategies. PrEP, pre-exposure prophylaxis; QALY, quality-adjusted life years. Zone 1 (areas above the red dashed line): Interventions located in zone 1 cost more than three times per capita gross domestic product per QALY gained, and are defined as not cost effective. Zone 2 (areas between the red dashed line and the blue dashed line): Interventions located in zone 2 cost between one and three times per capita gross domestic product per QALY gained, and are defined as cost effective [40, 41]. Zone 3 (areas below the blue dashed line): Interventions located in zone 3 cost less than per capita gross domestic product per QALY gained, and are defined as very cost effective [40, 41]. The black dashed line represents the optimal cost-effectiveness path.

The costs and health benefits (QALYs) for all intervention scenarios are shown in Table 1 and Fig. 3. Among all scenarios, the most cost-effective strategy was test-and-treat, which could prevent 57.9% (0.45 million) of the total new infections estimated under the status quo scenario, with an average CER of 1754 Int.$/QALY.

With PrEP effectiveness of 60%, the PrEP-only strategies for high-risk MSM (scenario 2–4) can prevent 0.09–0.20 million infections, 12.1–25.7% of the total new infections, yielding an average CER of 17,277–18,452 Int.$/QALY.

Compared to the base-case scenario, the combination strategies of PrEP and expanded VCT (scenario 5–7) could prevent 0.24–0.36 million new infections, 30.8–47.0% of the total new infections, at a cost of 13,835–18,110 Int.$ per QALY gained. With a combination of test-and-treat and PrEP for 75% of high-risk MSM (scenario 10), 0.58 million new infections (i.e., 75.2% of the total new infections) could be prevented, at a cost of 12,218 Int.$ per QALY gained. When resources are available, the optimal cost-effectiveness path is from test-and-treat to the combination strategy of test-and-treat and PrEP (25% of high-risk MSM), with an ICER of 72,545 Int.$/QALY; followed by the same combination strategy of test-and-treat and PrEP, but with higher PrEP coverage, costing 65,428–73,500 Int.$ per QALY gained depending on coverage level.

Table 1 also shows the saving in healthcare cost for all intervention scenarios compared to the base case. The test-and-treat strategy was the most cost-saving strategy, with a much greater saving in healthcare cost than the incremental costs. Among all combination strategies, the savings in healthcare costs can cover 63.7–100% of the incremental cost, and they can avert 0.08–0.13 million more infections compared to test-and-treat only strategy.

### Sensitivity analysis

The sensitivity ranges of the epidemiological impact and cost-effectiveness are also shown in Table 1. The test and treat strategy would avert 46.4–66.5% of the total new HIV infections, whereas PrEP covering 25%/50%/75% of high-risk MSM would avert 8.7–16.7%, 15.1–26.9%, and 19.6–33.3% of the total new infections, respectively. With a combination strategy of test-and-treat and PrEP (75% of high-risk MSM), 69.7–80.5% of the total new infections over 20 years would be prevented. All ten strategies (scenario 1–10) remain cost-effective compared to the base case under all ranges of sensitivity values (Table 1), costing less than three times per capita gross domestic product per QALY gained.

We also tested the robustness of our findings to changes in the assumption about PrEP effectiveness, which depends on PrEP adherence (Additional file 1 Figure S3 and Table S5 in the supplement). With a decrease in PrEP effectiveness from 60 to 30%, under the PrEP 25%/50%/75% high-risk MSM strategies (scenarios 2–4), the total number of new infections prevented will drop from 0.09–0.20 million to 0.05–0.12 million (12.1–25.7% vs. 7.0–14.9%, additional file 1 Figure S3 and Table S5); the cost per QALY gained will increase from 17,277–18,452 Int.$ to 29,902–30,414 Int.$ (Table S5 in the supplement). The results with an increase in PrEP effectiveness from 60 to 90% are described in the supplement.

In the analysis of risk compensation, it was assumed that all PrEP users in scenario 2–10 completely stopped using condoms with their sex partners. With an assumed PrEP effectiveness of 60%, the number of infections prevented will reduce by around 22.2–31.2% in scenarios 2–4 with risk compensation compared to the scenarios without risk compensation (Table S6 (c)), suggesting that risk compensation could be a significant hindrance to the impact of this strategy in situations where PrEP effectiveness is not ideal.

Finally, we modeled the impact of all interventions over a 30-year time horizon, shown in section 3.2 of the supplementary materials.

## Discussion

This study used a compartmental mathematical model to estimate the future trends of the HIV epidemic among MSM in China and to evaluate the cost-effectiveness of individual and combined biomedical interventions. The base case scenario estimated that without scale-up of current treatment strategies and implementation of new interventions the HIV epidemic will not be well controlled in MSM. The provision of PrEP to 25% of high-risk MSM in combination with a fully implemented test-and-treat strategy could prevent more than 0.5 million new HIV infections by 2037. With the combined strategy of test-and-treat and PrEP among high-risk MSM it is possible to achieve HIV elimination by 2033. All individual and combined biomedical interventions were shown to be cost-effective, and two strategies were also shown to be cost-saving.

UNAIDS proposed the ambitious 90–90-90 targets to end the HIV epidemic by 2030. However, in this study, we found that the 90–90-90 strategy alone may not be sufficient to eliminate HIV transmission. Under this strategy, HIV incidence at 2037 is estimated to be 2.4 per 1000 person-years, which is much higher than the incidence threshold for HIV elimination. This result confirms findings from other studies [15, 27, 28] and shows that novel HIV prevention strategies are greatly warranted among MSM in China.

This study also confirms other findings about the effectiveness and cost-effectiveness of PrEP strategies among MSM [27, 29–31], extends these findings to a middle-income setting and compares them against test-and-treat strategies. This study also confirms a recent finding about the epidemiological impact and effectiveness of PrEP strategies among Chinese MSM [32]. In this study, we further investigated the conditions for HIV elimination and tested the impact of PrEP adherence and risk compensation on the main findings. Both of these studies show the importance of scaling up existing strategies and implementing novel intervention strategies in China. Our effectiveness and cost-effectiveness estimates may be underestimated because: 1) the PrEP strategy is a comprehensive programme, rather than solely biomedical. Although not modeled in this study, the PrEP programme also includes components of regular HIV testing, behavioral counselling, motivational interviewing and risk reduction, which could lead to further gains in health benefits; 2) in this study, we only analyzed a daily PrEP strategy, but an on-demand strategy would reduce the average number of days that the participants need to take PrEP, lowering the cost of PrEP interventions and significantly improving their acceptability; and 3) the patents for PrEP drugs expired in December 2017 [30], leading to potential future price reductions that could not be incorporated into the cost estimates for this study.

Previously published clinical trials [12, 33] have shown that the protective effects of PrEP relied heavily on drug adherence, which will likely be lower in actual implementation than in trials. In this study we assumed a PrEP effectiveness of 60%, which reflected the adherence rates in PrEP demonstration studies in China [23]. In the scenario-based sensitivity analysis, we tested possible effectiveness of both increased (90%) and decreased (30%) adherence rates. Even under the worst adherence scenario of 30% PrEP effectiveness, the PrEP only strategies can still prevent 7.0–14.9% of HIV infections over a 20 year time horizon. This shows that, while efforts to improve adherence will be important in practical implementation of PrEP in China, failure to achieve trial-level adherence will not fundamentally undermine the effectiveness of the policy itself.

Another concern about PrEP is risk compensation. A recent meta-analysis found evidence of an increase in condomless sex and STI prevalence among PrEP users [34]. One study found that 35% of MSM in China were more likely to have unprotected sex while taking PrEP [35]. Our risk-compensation analyses also found that when PrEP adherence is low, its effectiveness is sensitive to risk compensation. However, with good PrEP adherence (90%), even a 100% risk compensation in condom use would not affect the effectiveness of the intervention for HIV prevention. Therefore, if significant levels of risk compensation are expected amongst the target population, intensive efforts at improving adherence will be needed to realize the full benefits of the PrEP policy. Furthermore, we did not model multiple risk-compensation situations, in which MSM receiving PrEP increase both unsafe sex and number of partners. An appropriately designed and well-implemented demonstration study is needed in China to fully answer these questions, but the possibility of risk compensation should not be taken as a reason not to consider implementing PrEP in China, as our modeling shows that only the most extreme forms of risk compensation will undermine the strategy, and even then it will likely still realize some benefits. However, it is always important to monitor risk compensation among users in the context of PrEP scale-up to assess the impact of PrEP on the sexual health (including HIV and other STI) of users and to inform preventive strategies.

Our study has several limitations. First, heterosexual transmission was not discussed in our model, although 17–35% of Chinese MSM are married [36]. Second, we only targeted up to 75% of high-risk MSM for PrEP. Targeting a larger percentage or expanding to low-risk MSM would lead to a higher impact on the HIV epidemic but a reduced cost-effectiveness. We chose the 75% coverage strategy because awareness of PrEP is low among Chinese MSM [37], and a targeted program aimed at high-risk MSM could be more achievable and feasible. Third, we also did not include false-positive results, resistance to ART, and adverse events of PrEP in our model, but unless extreme negative events happen during the intervention period, their effects on the impact of interventions are not likely to be substantial. Fourth, three of our PrEP scenarios (scenarios 5–7) include the assumption of high rates of voluntary counselling and testing, that might be infeasible in the current environment in China. However, to account for this we also considered three basic scenarios (scenarios 2–4) where PrEP was offered to high-risk MSM, but these MSM did not increase their testing rates to high levels. Even in these more realistic scenarios, we found PrEP was effective. Finally, our study used commonly-accepted cost-effectiveness thresholds of 1-3x per capita GDP for identifying which strategies would be cost-effective, but recent research has shown that these thresholds may not be appropriate for low- and middle-income countries, where use of these thresholds may lead to adoption of lower priority interventions [38]. Using a proposed alternative threshold for China of approximately 50% of GDP [39] would potentially change our conclusion regarding the cost-effectiveness of many of the interventions we considered, but even under this much stricter experimental alternative cost-effectiveness threshold, the test-and-treat strategy, and test-and-treat with 25% PrEP, remain cost-effective. This shows the enormous public health potential of improved HIV interventions in China.

## Conclusion

Our findings suggest that both test-and-treat and PrEP can be effective tools for HIV prevention among MSM, and are predicted to be cost-effective even with poor PrEP effectiveness. HIV elimination is achievable in the next 20 years with a combination of these biomedical interventions. PrEP for HIV prevention should be considered for implementation among high-risk MSM in China.

## Additional files

Additional file 1: Supplementary Appendix. A complete description of the mathematical methods used in development of the model. (DOCX 5387 kb)

## Abbreviations

ART: Antiretroviral therapy
CER: Cost-effectiveness ratio
GDP: Gross domestic product
HIV: Human Immunodeficiency Virus
ICER: Incremental cost-effectiveness ratio
MSM: Men who have sex with men
ODE: Ordinary differential equation
PLWH: People living with HIV
PrEP: Pre-exposure prophylaxis
QALY: Quality-adjusted life year
RCTs: Randomized controlled trials
UNAIDS: Joint United Nations Programme on HIV/AIDS
USA: United States of America
WHO: World Health Organization
