# Supplementary material for: A mathematical model of biomedical interventions for HIV prevention among men who have sex with men in China
Source: BMC Infect Dis. 2018 Nov 28;18:600. doi: 10.1186/s12879-018-3516-8 (PMC6263536; doi:10.1186/s12879-018-3516-8)
Supplement: Supplementary file 1 — Supplementary Appendix. A complete description of the mathematical methods used in development of the model. (DOCX 5387 kb) [file 12879_2018_3516_MOESM1_ESM.docx]

**Supplements**

# Contents

1. Contents 2

2. Supplementary methods 4

2.1. Epidemic model framework 4

2.1.1. Main model structure 5

2.1.2. Key model parameters 9

2.2. Model equations 11

2.2.1. Differential equations 11

2.2.2. Entry rates, maturation rates and initial values for the populations 13

2.2.3. Heterogeneity in risk behavior 14

2.2.4. Transmission forces 17

2.3. Intervention types and assumptions 23

2.4. Model outputs 24

2.4.1. Epidemiological Outcomes 24

2.4.2. Economic Outcomes 25

2.5. Model simulation 26

2.6. Model calibration 27

3. Supplementary results 30

3.1. Sensitivity analysis 30

3.1.1. PrEP effectiveness 30

3.1.2. Risk compensation 31

3.2. Main results with time horizon of 30 years 43

3.2.1. HIV epidemic projections 43

3.2.2. Impact of biomedical interventions 43

4. References 49

# Supplementary methods

This document describes the basic structure of the model, introduces the model equations and provides details of the values used for all input parameters. It also gives details of the calibration method used to assess the model fit and select candidate models for the final cost-effectiveness analysis.

## Epidemic model framework

This study used a compartmental, deterministic model that was calibrated against observed prevalence data using a deviance criterion and projected forward using a difference equation method. The model structure is designed to reflect the natural history of HIV and the current policy framework for HIV interventions. Parameters for the model were obtained from published literature and publicly available data sources, and equations governing risk behavior and population mixing followed standard practice for sexually transmitted infections.

The compartmental structure was applied to two risk groups – low and high risk men – that were allowed to interact with each other according to an established probabilistic framework that determined the force of infection at any time point. Difference equations were solved using a monthly time step, but model results are reported at the annual level consistent with past studies and the available calibration data. The model was run on a population of Chinese MSM aged 15-64.

### Main model structure

Figure S1 shows the compartmental structure of the model, with each box showing a population group and the arrows describing flows between population groups. Greek symbols above or next to the arrows show transition rates. Arrows pointing out of a box and not terminating on a box represent mortality from that population group, while the arrow pointing into box 1 indicates new entrants to the population. Note that due to population ageing the exit rates incorporate mortality and population maturation into the elderly population.

For example, box 7 in Figure S1 represents those members of the MSM population who are living with asymptomatic HIV, with a CD4 count over 500 cells/mm3, who has not yet been identified as HIV positive and so remains not in treatment and making no behavioral changes consistent with identification as a person living with HIV (PLWH). People enter this box at a rate $\theta_{4}$ from box 4, which contains newly-infected people with acute HIV infection who do not know their serostatus. They leave this box either by mortality/maturation (composed of HIV-related mortality $\mu_{7}$ and background mortality/maturation $b_{7}$); by being tested for HIV, learning their serostatus, and moving to box 8 with rate $\psi_{7}$ ; or by progressing in their HIV symptoms to the asymptomatic stage with CD4 count between 200 and 500 cells/mm3, in box 10, at rate $\theta_{7}$. All rates are converted to transition probabilities for the purpose of the calculation of monthly difference equations. The rates are combined in a single differential equation describing the total flow into and out of each cell, providing a total of 15 differential equations in each risk group.


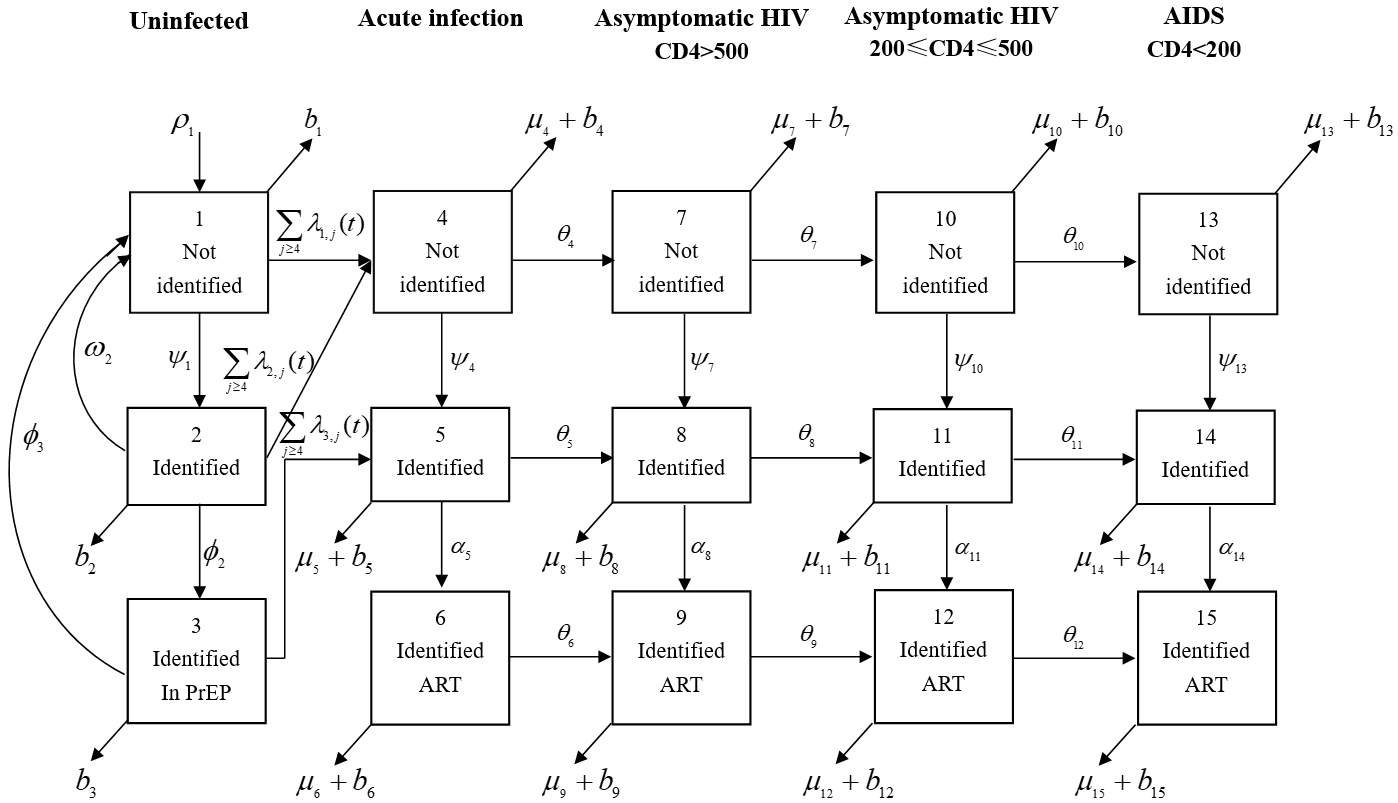


Figure S1 HIV transmission compartmental model structure

Table S1 provides the meaning of all symbols in Figure S1. The key dynamically varying rate is the force of infection, $\sum_{j\geq4} \lambda_{k,j}^{i}(t)$, which is determined by the interaction of population sizes and mixing rates (see sections 2.2.3 and 2.2.4). Specific values chosen for these rates, and the sources from which those numbers were drawn, are shown in Table S2. Note that in this model all transition rates are treated as equal between low- and high-risk men, with the primary driver of different rates of infection between these two groups determined by their sexual behavior variables, which enter the model through the calculation of the force of infection.

Table S1 Summary and description of model variables

| Variables/ Symbols | Definition |
| --- | --- |
| Demographic characteristics | |
| $X_{i,j}$ | Number of people in risk group *i* with status *j* |
| $b_{j}^{i}$ | Annual maturation rate |
| $\mu_{j}^{i}$ | Annual mortality rate due to HIV/AIDS |
| $\rho_{1}^{i}$ | Annual entry rate |
| Homosexual transmission | |
| $\sigma^{Z}$ | Annual transmission probability per partnership from male to male, where z= asymptomatic HIV (CD4>500), asymptomatic HIV (200≤CD4≤500), and AIDS (CD4<200) |
| $n_{1}$ | Annual same-sex partners of LRMSM |
| $u_{1}$ | Condom use with same-sex partners of LRMSM, percent |
| $n_{2}$ | Annual same-sex partners of HRMSM |
| $u_{2}$ | Condom use with same-sex partners of HRMSM, percent |
| $\kappa$ | Condom effectiveness |
| Voluntary Counseling and Testing | |
| $\psi_{j}^{i}$ | Fraction of population tested in past 12 months for risk group *i* with status *j*, percent |
| $1/\omega_{2}^{i}$ | Average duration (years) that uninfected individuals remain identified after testing in risk group *i* |
| $r_{1}$ | Reduction in sexual behavior among persons identified as HIV-positive, percent |
| $r_{1}^{'}$ | Reduction in sexual behavior among people with AIDS, percent |
| ART Treatment | |
| $\alpha_{j}^{i}$ | Fraction starting ART in risk group *i* with status *j* |
| $r_{2}$ | Reduction in sexual infectivity due to ART, percent |
| PrEP program | |
|  | Rate at which susceptible in risk group *i* start taking PrEP |
| $\omega_{3}^{i}$ | Rate of PrEP dropout per person per year in risk group *i*. |
| $r_{3}$ | Risk reduction of PrEP against HIV infection, percent |
| Cost-effectiveness | |
| $q_{j}^{i}$ | Quality-of-life adjustment of individuals in risk group *i* with status *j* |
| $c_{j}^{i}$ | Annual ART cost per person |
| $c'_{j}^{i}$ | Annual health care cost per individual in risk group *i* with status *j* |
| $c_{neg}$ | VCT cost per HIV-negative person, including cost of counseling, ELISA test |
| $c_{pos}$ | VCT cost per HIV-positive person, including cost of counseling, ELISA test and Western blot test |
| $c_{PrEP}$ | Annual PrEP cost per person, including PrEP drug and PrEP clinic costs. |
| r | Discount rate |
| Others | |
| $\theta_{j}^{i}$ | HIV disease progression rate for individuals in risk group *i* with status *j* |
| $\sum_{j\geq4} \lambda_{k,j}^{i}(t)$ | Transmission forces for each risk groups *i* |

### Key model parameters

Key model parameters are shown in Table S2 with their description and published source.

Table S2 Key model parameters

| Variable | Value | References |
| --- | --- | --- |
| *Demographic characteristics* |  |  |
| Annual mortality rate (background) | 0.00711 | Calculated, ^1^ |
| Annual mortality rate (due to HIV/AIDS) |  |  |
| Asymptomatic (CD4>500) | 0.02 | 2 |
| Asymptomatic (200≤CD4≤500) | 0.063 | 2 |
| AIDS (CD4<200) | 0.22 | 3,4 |
| Asymptomatic with ART | 0.05 | 2,5 |
| AIDS with ART | 0.075 | 5 |
| Annual maturation rate | 0.022 | Calculated, ^1^ |
| Annual entry rate | 0.028 | Calculated, ^1^ |
| Initial population (aged 15-64) |  |  |
| Low-risk MSM | 2,900,000 | Calculated, ^6^ |
| High-risk MSM | 725,000 | Calculated, ^6^ |
| Initial HIV prevalence among MSM in 2005 (aged 15-64), % | 3.4 | 7,8 |
| *Sexual transmission* |  |  |
| Transmission probability per partnership (male to male) |  |  |
| Acute | 0.21 | 9 |
| Asymptomatic (CD4>500) | 0.03 | 10-13 |
| Asymptomatic (200≤CD4≤500) | 0.045 | 10-13 |
| AIDS (CD4<200) | 0.12 | 10-13 |
| Annual same-sex partners |  |  |
| Average for all MSM | 5 | 14 |
| High-risk MSM | 15 | Assumed |
| Low-risk MSM | 2.6 | Calculated^#^ |
| Condom use with same-sex partners, % |  |  |
| MSM | 36% | 15,16 |
| Condom effectiveness | 0.9 | 17-19 |
| *HIV VCT* |  |  |
| Proportion of population tested in past 12 months, % | 37% | 20 |
| Reduction in sexual behavior among persons identified as HIV-positive, % | 20% | 21 |
| Reduction in sexual behavior among AIDS patients, % | 90% | Assumed |
| *ART* |  |  |
| Proportion starting ART at CD4 cell count of 500 or less | 30% | 20 |
| Reduction in sexual infectivity due to ART, % | 90% | 22-24 |
| *Disease stage duration, years* |  |  |
| Acute stage | 0.25 | 25 |
| Asymptomatic (CD4>500) | 1.19 | 25 |
| Asymptomatic (200≤CD4≤500) | 6.74 | 25 |
| *Quality of life multipliers* |  |  |
| HIV negative/on PrEP | 1.00 |  |
| HIV positive (CD4>500) | 0.94 | 26,27 |
| HIV positive (200≤CD4≤500) | 0.85 |  |
| AIDS (CD4<200) | 0.73 | 26-29 |
| HIV positive (CD4>500) with ART | 0.95 | 26,27 |
| HIV positive (200≤CD4≤500) with ART | 0.90 |  |
| AIDS (CD4<200) with ART | 0.82 | 19,27,29,30 |
| *Cost, 2017 Int.$* |  |  |
| Annual HIV-related health care cost |  |  |
| HIV positive (CD4>500) | 2,624 | Interpolated^&^ |
| HIV positive (200≤CD4≤500) | 4,406 | 31 |
| AIDS (CD4<200) | 13,585 | 31 |
| HIV positive (CD4>500) with ART | 2,624 | Interpolated^&^ |
| HIV positive (200≤CD4≤500) with ART | 3,928 | Interpolated^&^ |
| AIDS (CD4<200) with ART | 6,182 | Interpolated^&^ |
| Annual non-HIV related health care cost | 764 | 32 |
| Annual cost of ART | 6,540 | 33 |
| Cost of HIV ELISA antibody test | 25 | 34 |
| Cost of confirmatory western blot test | 85 | 34 |
| Cost of behavior counseling, | 28 | 35 |
| Annual discount rate, % | 3 |  |
| Annual cost of PrEP (including PrEP drug cost and clinics cost) | 6,909 | Estimated^ |

^#^This is a derived value, calculated in order to balance the total number of partnerships formed by low-risk MSM and high-risk MSM;

^&^Calculated by multiplying health care costs of untreated asymptomatic HIV/AIDS by the ratios of the cost of this disease stage with other stages in published papers^36^;

^Estimated based on the domestic price of Truvada.

## Model equations

### Differential equations

The system of 15 ordinary differential equations (ODEs) describing inter-compartmental flow are shown below. This set of ODEs is the same for both risk groups (high-risk and low-risk MSM, so the complete model comprises 30 equations. We coded these equations, initial values and parameters using MATLAB R2016b. The 15 equations for each risk group are:

| $\frac{dX_{i,1}}{dt}=\rho_{1}^{i}\sum_{\forall j} X_{i,j}+\omega_{2}^{i}X_{i,2}+\varphi_{3}^{i}X_{i,3}-\left(\sum_{j\geq4} \lambda_{1,j}^{i}(t)\right)X_{i,1}-(\psi_{1}^{i}+b_{1}^{i})X_{i,1}$ | (1) |
| --- | --- |
| $\frac{dX_{i,2}}{dt}=\psi_{1}^{i}X_{i,1}-\left(\sum_{j\geq4} \lambda_{2,j}^{i}(t)\right)X_{i,2}-(\omega_{2}^{i}+\varphi_{2}^{i}+b_{2}^{i})X_{i,2}$ | (2) |
| $\frac{dX_{i,3}}{dt}=\varphi_{2}^{i}X_{i,2}-\left(\sum_{j\geq4} \lambda_{3,j}^{i}(t)\right)X_{i,3}-(\varphi_{3}^{i}+b_{3}^{i})X_{i,3}$ | (3) |
| $\frac{dX_{i,4}}{dt}=\left(\sum_{j\geq4} \lambda_{1,j}^{i}(t)\right)X_{i,1}+\left(\sum_{j\geq4} \lambda_{2,j}^{i}(t)\right)X_{i,2}-(\psi_{4}^{i}+\theta_{4}^{i}+\mu_{4}^{i}+b_{4}^{i})X_{i,4}$ | (4) |
| $\frac{dX_{i,5}}{dt}=\left(\sum_{j\geq4} \lambda_{3,j}^{i}(t)\right)X_{i,3}+\psi_{4}^{i}X_{i,4}-(\theta_{5}^{i}+\alpha_{5}^{i}+\mu_{5}^{i}+b_{5}^{i})X_{i,5}$ | (5) |
| $\frac{dX_{i,6}}{dt}=\alpha_{5}^{i}X_{i,5}-(\theta_{6}^{i}+\mu_{6}^{i}+b_{6}^{i})X_{i,6}$ | (6) |
| $\frac{dX_{i,7}}{dt}=\theta_{4}^{i}X_{i,4}-(\psi_{7}^{i}+\theta_{7}^{i}+\mu_{7}^{i}+b_{7}^{i})X_{i,7}$ | (7) |
| $\frac{dX_{i,8}}{dt}=\theta_{5}^{i}X_{i,5}+\psi_{7}^{i}X_{i,7}-(\theta_{8}^{i}+\alpha_{8}^{i}+\mu_{8}^{i}+b_{8}^{i})X_{i,8}$ | (8) |
| $\frac{dX_{i,9}}{dt}=\theta_{6}^{i}X_{i,6}+\alpha_{8}^{i}X_{i,8}-(\theta_{9}^{i}+\mu_{9}^{i}+b_{9}^{i})X_{i,9}$ | (9) |
| $\frac{dX_{i,10}}{dt}=\theta_{7}^{i}X_{i,7}-(\psi_{10}^{i}+\theta_{10}^{i}+\mu_{10}^{i}+b_{10}^{i})X_{i,10}$ | (10) |
| $\frac{dX_{i,11}}{dt}=\theta_{8}^{i}X_{i,8}+\psi_{10}^{i}X_{i,10}-(\theta_{11}^{i}+\alpha_{11}^{i}+\mu_{11}^{i}+b_{11}^{i})X_{i,11}$ | (11) |
| $\frac{dX_{i,12}}{dt}=\theta_{9}^{i}X_{i,9}+\alpha_{11}^{i}X_{i,11}-(\theta_{12}^{i}+\mu_{12}^{i}+b_{12}^{i})X_{i,12}$ | (12) |
| $\frac{dX_{i,13}}{dt}=\theta_{10}^{i}X_{i,10}-(\psi_{13}^{i}+\mu_{13}^{i}+b_{13}^{i})X_{i,13}$ | (13) |
| $\frac{dX_{i,14}}{dt}=\theta_{11}^{i}X_{i,11}+\psi_{13}^{i}X_{i,13}-(\alpha_{14}^{i}+\mu_{14}^{i}+b_{14}^{i})X_{i,14}$ | (14) |
| $\frac{dX_{i,15}}{dt}=\theta_{12}^{i}X_{i,12}+\alpha_{14}^{i}X_{i,14}-(\mu_{15}^{i}+b_{15}^{i})X_{i,15}$ | (15) |

Where i indexes the risk groups (1: low-risk MSM, LRMSM and 2: high-risk MSM, HRMSM). j corresponds to the 15 compartments reflecting HIV progression (1: unidentified uninfected, 2: identified uninfected, 3: identified uninfected with PrEP, 4: unidentified acute infection, 5: identified acute infection, 6: identified acute infection with ART, 7: unidentified asymptomatic (CD4>500), 8: identified asymptomatic (CD4>500), 9: identified asymptomatic (CD4>500) with ART, 10: unidentified asymptomatic (200≤CD4≤500), 11: identified asymptomatic (200≤CD4≤500), 12: identified asymptomatic (200≤CD4≤500) with ART, 13: unidentified AIDS, 14: identified AIDS, 15: identified AIDS with ART). Parameter values are shown in Table S2.

### Entry rates, maturation rates and initial values for the populations

Our target population is men who have sex with men aged 15-64 years old. The entry rate is defined as the rate at which people enter the target group, derived from existing demographic data for China. The maturation rate is the sum of the background mortality rate and the rate of aging. We assume that LRMSM and HRMSM have the same entry and maturation rates as the general population.

Entry rates:

| $\rho\text{=}-\ln(1-\frac{\text{15 years old population}}{\text{ 15-64 years old population}})+\text{growth rate}$ | (16) |
| --- | --- |

Maturation rates:

| $\text{b=}-\ln(1-\frac{\text{64 years old population}}{\text{ 15-64 years old population}})+\text{mortality rate}$ | (17) |
| --- | --- |

Initial values for the population of LRMSM and HRMSM are shown in Table S3, calculated from population size and prevalence information in existing studies^7,8,37^.

Table S3 Initial values for the population of LRMSM and HRMSM groups

|  | LRMSM | HRMSM |
| --- | --- | --- |
| X1 | 1781096 | 426147 |
| X2 | 1046040 | 250277 |
| X3 | 0 | 0 |
| X4 | 2295 | 1530 |
| X5 | 1347 | 898 |
| X6 | 0 | 0 |
| X7 | 6701 | 4467 |
| X8 | 3936 | 2624 |
| X9 | 0 | 0 |
| X10 | 21069 | 14046 |
| X11 | 8661 | 5774 |
| X12 | 3712 | 2474 |
| X13 | 15836 | 10557 |
| X14 | 6510 | 4340 |
| X15 | 2790 | 1860 |

### Heterogeneity in risk behavior

This model uses two populations – high risk and low risk MSM – that are defined by their different sexual partnership numbers. Members of both populations are able to have sexual interactions with members of the other population, but consistent with general principles for modeling sexually transmissible infections with two populations,^38^ it is necessary to assume a certain level of mixing between the populations. Given a mixing parameter $\epsilon$, we defined the proportion of a LRMSM’s partnerships that are with LRMSM as:

| $\eta_{1,1}\text{=}(1-\varepsilon)+\varepsilon\frac{TP1}{TP1+TP2}$ | (18) |
| --- | --- |

The proportion of a LRMSM’s partnerships that are with HRMSM is:

| $\eta_{1,2}\text{=}\varepsilon\frac{TP2}{TP1+TP2}$ | (19) |
| --- | --- |

The proportion of a HRMSM’s partnerships that are with LRMSM is:

| $\eta_{2,1}\text{=}\varepsilon\frac{TP1}{TP1+TP2}$ | (20) |
| --- | --- |

The proportion of a HRMSM’s partnerships that are with HRMSM is:

| $\eta_{2,2}\text{=}(1-\varepsilon)+\varepsilon\frac{TP2}{TP1+TP2}$ | (21) |
| --- | --- |

Here, $\varepsilon$defines the degree of randomness of the partnership process, with $\varepsilon$=0 defining completely assortative mixing, where people only have sexual encounters with their own group, while $\varepsilon$=1 defines a totally random mixing pattern.

Having defined the mixing pattern, we now define the total number of partnerships for high- and low-risk MSM. This is necessary in order to obtain an average number of partnerships for the entire population that is consistent with published estimates for the MSM population as a whole, and as part of the calculation of the force of infection.

$TP1$is the total partnerships for LRMSM (*i*=1, *j*=1~15):

$TP1=\sum_{i=1} \left[(\sum_{j=1,2,3,4,7,10} X_{i,j})n_{i}+(\sum_{j=5,6,8,9,11,12} X_{i,j})n_{i}(1-r_{1})+(\sum_{j=13,14,15} X_{i,j})n_{i}(1-r_{1}')\right]$ (22)

$TP2$is the total partnerships for HRMSM (*i*=2, *j*=1~15):

$TP2=\sum_{i=2} \left[(\sum_{j=1,2,3,4,7,10} X_{i,j})n_{i}+(\sum_{j=5,6,8,9,11,12} X_{i,j})n_{i}(1-r_{1})+(\sum_{j=13,14,15} X_{i,j})n_{i}(1-r_{1}')\right]$ (23)

These figures are incorporated into the calculation of the force of infection, which is described in section 2.2.4.

### Transmission forces

The force of infection is determined as the probability that a single sexual partnership is infectious, for any given person who is HIV negative and engaging in unprotected sex with a member of any other HIV positive group. This requires estimation of the probability that any sexual contact is with a member of one of the population groups capable of transmitting HIV infection. The specific method for calculating these details is outlined here.

#### Common transmission formulae

Susceptible individuals can become infected though homosexual contact with HIV-positive HRMSM or HIV-positive LRMSM.

The probability that MSM without PrEP are not infected by HIV-positive LRMSM in compartment j, through one homosexual partnership, $N_{-}LRMSM_{j}$ (j=4~15), is (for each compartment in turn):

$$N_{-}LRMSM_{4}=\left[1-\left(\frac{X_{1,4}n_{1}}{TP1}\sigma^{acute}\right)\right];N_{-}LRMSM_{5}=\left[1-\left(\frac{X_{1,5}n_{1}(1-r_{1})}{TP1}\sigma^{acute}\right)\right];N_{-}LRMSM_{6}=\left[1-\left(\frac{X_{1,6}n_{1}(1-r_{1})}{TP1}\sigma^{acute}(1-r_{2})\right)\right];$$

$N_{-}LRMSM_{7}=\left[1-\left(\frac{X_{1,7}n_{1}}{TP1}\sigma^{asym}\right)\right];N_{-}LRMSM_{8}=\left[1-\left(\frac{X_{1,8}n_{1}(1-r_{1})}{TP1}\sigma^{asym}\right)\right];N_{-}LRMSM_{9}=\left[1-\left(\frac{X_{1,9}n_{1}(1-r_{1})}{TP1}\sigma^{asym}(1-r_{2})\right)\right];$ (24)

$$N_{-}LRMSM_{10}=\left[1-\left(\frac{X_{1,10}n_{1}}{TP1}\sigma^{asym}\right)\right];N_{-}LRMSM_{11}=\left[1-\left(\frac{X_{1,11}n_{1}(1-r_{1})}{TP1}\sigma^{asym}\right)\right];N_{-}LRMSM_{12}=\left[1-\left(\frac{X_{1,12}n_{1}(1-r_{1})}{TP1}\sigma^{asym}(1-r_{2})\right)\right];$$

$$N_{-}LRMSM_{13}=\left[1-\left(\frac{X_{1,13}n_{1}(1-r_{1}^{'})}{TP1}\sigma^{AIDS}\right)\right];N_{-}LRMSM_{14}=\left[1-\left(\frac{X_{1,14}n_{1}(1-r_{1}^{'})}{TP1}\sigma^{AIDS}\right)\right];N_{-}LRMSM_{15}=\left[1-\left(\frac{X_{1,15}n_{1}(1-r_{1}^{'})}{TP1}\sigma^{AIDS}(1-r_{2})\right)\right]$$

Where, $TP1$ is the total partnerships for LRMSM, defined in section 2.2.3 above.

The probability that MSM with PrEP are not infected during sexual contact with HIV-positive LRMSM in compartment j, through one homosexual contact, $N_{-}prepLRMSM_{j}$ (j=4~15), is:

$$N_{-}prepLRMSM_{4}=\left[1-\left(\frac{X_{1,4}n_{1}}{TP1}\sigma^{acute}(1-r_{3})\right)\right];N_{-}prepLRMSM_{5}=\left[1-\left(\frac{X_{1,5}n_{1}(1-r_{1})}{TP1}\sigma^{acute}(1-r_{3})\right)\right];N_{-}prepLRMSM_{6}=\left[1-\left(\frac{X_{1,6}n_{1}(1-r_{1})}{TP1}\sigma^{acute}(1-r_{2})(1-r_{3})\right)\right];$$

$$N_{-}prepLRMSM_{7}=\left[1-\left(\frac{X_{1,7}n_{1}}{TP1}\sigma^{asym}(1-r_{3})\right)\right];N_{-}prepLRMSM_{8}=\left[1-\left(\frac{X_{1,8}n_{1}(1-r_{1})}{TP1}\sigma^{asym}(1-r_{3})\right)\right];N_{-}prepLRMSM_{9}=\left[1-\left(\frac{X_{1,9}n_{1}(1-r_{1})}{TP1}\sigma^{asym}(1-r_{2})(1-r_{3})\right)\right]$$

$N_{-}prepLRMSM_{10}=\left[1-\left(\frac{X_{1,10}n_{1}}{TP1}\sigma^{asym}(1-r_{3})\right)\right];N_{-}prepLRMSM_{11}=\left[1-\left(\frac{X_{1,11}n_{1}(1-r_{1})}{TP1}\sigma^{asym}(1-r_{3})\right)\right];N_{-}prepLRMSM_{12}=\left[1-\left(\frac{X_{1,12}n_{1}(1-r_{1})}{TP1}\sigma^{asym}(1-r_{2})(1-r_{3})\right)\right];$ $N_{-}prepLRMSM_{13}=\left[1-\left(\frac{X_{1,13}n_{1}(1-r_{1}^{'})}{TP1}\sigma^{AIDS}(1-r_{3})\right)\right];N_{-}prepLRMSM_{14}=\left[1-\left(\frac{X_{1,14}n_{1}(1-r_{1}^{'})}{TP1}\sigma^{AIDS}(1-r_{3})\right)\right];N_{-}prepLRMSM_{15}=\left[1-\left(\frac{X_{1,15}n_{1}(1-r_{1}^{'})}{TP1}\sigma^{AIDS}(1-r_{2})(1-r_{3})\right)\right]$ (25)

Again, $TP1$ is the total partnerships for LRMSM.

By symmetry, the probability that men are not infected by HIV-positive HRMSM in compartment j, though one homosexual contact, $N_{-}HRMSM_{j}$ (j=4~15), is:

$$N_{-}HRMSM_{4}=\left[1-\left(\frac{X_{2,4}n_{2}}{TP2}\sigma^{acute}\right)\right];N_{-}HRMSM_{5}=\left[1-\left(\frac{X_{2,5}n_{2}(1-r_{1})}{TP2}\sigma^{acute}\right)\right];N_{-}HRMSM_{6}=\left[1-\left(\frac{X_{2,6}n_{2}(1-r_{1})}{TP2}\sigma^{acute}(1-r_{2})\right)\right];$$

$$N_{-}HRMSM_{7}=\left[1-\left(\frac{X_{2,7}n_{2}}{TP2}\sigma^{asym}\right)\right];N_{-}HRMSM_{8}=\left[1-\left(\frac{X_{2,8}n_{2}(1-r_{1})}{TP2}\sigma^{asym}\right)\right];N_{-}HRMSM_{9}=\left[1-\left(\frac{X_{2,9}n_{2}(1-r_{1})}{TP2}\sigma^{asym}(1-r_{2})\right)\right]$$

$N_{-}HRMSM_{10}=\left[1-\left(\frac{X_{2,10}n_{2}}{TP2}\sigma^{asym}\right)\right];N_{-}HRMSM_{11}=\left[1-\left(\frac{X_{2,11}n_{2}(1-r_{1})}{TP2}\sigma^{asym}\right)\right];N_{-}HRMSM_{12}=\left[1-\left(\frac{X_{2,12}n_{2}(1-r_{1})}{TP2}\sigma^{asym}(1-r_{2})\right)\right];$ (26)

$$N_{-}HRMSM_{13}=\left[1-\left(\frac{X_{2,13}n_{2}(1-r_{1}^{'})}{TP2}\sigma^{AIDS}\right)\right];N_{-}HRMSM_{14}=\left[1-\left(\frac{X_{2,14}n_{2}(1-r_{1}^{'})}{TP2}\sigma^{AIDS}\right)\right];N_{-}HRMSM_{15}=\left[1-\left(\frac{X_{2,15}n_{2}(1-r_{1}^{'})}{TP2}\sigma^{AIDS}(1-r_{2})\right)\right]$$

Where, $TP2$ is the total partnerships for HRMSM.

Similarly, the probability that men with PrEP are not infected by HIV-positive HRMSM in compartment j, though one homosexual contact, $N_{-}prepHRMSM_{j}$ (j=4~15) is:

$N_{-}prepHRMSM_{4}=\left[1-\left(\frac{X_{2,4}n_{2}}{TP2}\sigma^{acute}(1-r_{3})\right)\right];N_{-}prepHRMSM_{5}=\left[1-\left(\frac{X_{2,5}n_{2}(1-r_{1})}{TP2}\sigma^{acute}(1-r_{3})\right)\right];N_{-}prepHRMSM_{6}=\left[1-\left(\frac{X_{2,6}n_{2}(1-r_{1})}{TP2}\sigma^{acute}(1-r_{2})(1-r_{3})\right)\right];$ $N_{-}prepHRMSM_{7}=\left[1-\left(\frac{X_{2,7}n_{2}}{TP2}\sigma^{asym}(1-r_{3})\right)\right];N_{-}prepHRMSM_{8}=\left[1-\left(\frac{X_{2,8}n_{2}(1-r_{1})}{TP2}\sigma^{asym}(1-r_{3})\right)\right];N_{-}prepHRMSM_{9}=\left[1-\left(\frac{X_{2,9}n_{2}(1-r_{1})}{TP2}\sigma^{asym}(1-r_{2})(1-r_{3})\right)\right];$ $N_{-}prepHRMSM_{10}=\left[1-\left(\frac{X_{2,10}n_{2}}{TP2}\sigma^{asym}(1-r_{3})\right)\right];N_{-}prepHRMSM_{11}=\left[1-\left(\frac{X_{2,11}n_{2}(1-r_{1})}{TP2}\sigma^{asym}(1-r_{3})\right)\right];N_{-}prepHRMSM_{12}=\left[1-\left(\frac{X_{2,12}n_{2}(1-r_{1})}{TP2}\sigma^{asym}(1-r_{2})(1-r_{3})\right)\right];$ $N_{-}prepHRMSM_{13}=\left[1-\left(\frac{X_{2,13}n_{2}(1-r_{1}^{'})}{TP2}\sigma^{AIDS}(1-r_{3})\right)\right];N_{-}prepHRMSM_{14}=\left[1-\left(\frac{X_{2,14}n_{2}(1-r_{1}^{'})}{TP2}\sigma^{AIDS}(1-r_{3})\right)\right];N_{-}prepHRMSM_{15}=\left[1-\left(\frac{X_{2,15}n_{2}(1-r_{1}^{'})}{TP2}\sigma^{AIDS}(1-r_{2})(1-r_{3})\right)\right]$ (27)

Again, $TP2$ is the total partnerships for HRMSM.

#### Transmission rates

Transmission forces$\sum_{j\geq4} \lambda_{1,j}^{i}(t),\sum_{j\geq4} \lambda_{2,j}^{i}(t),and\sum_{j\geq4} \lambda_{3,j}^{i}(t)$, quoted in equations (1) to (10), for LRMSM and HRMSM are defined in terms of the probabilities defined above.

Transmission forces for LRMSM are (for LRMSM not in PrEP and in PrEP, respectively):

| $\sum_{j\geq4} \lambda_{1,j}^{1}(t)=\sum_{j\geq4} \lambda_{2,j}^{1}(t)=\sum_{j\geq4} \left\{\left.1-N_{-}LRMSM_{j}^{\eta_{1,1}n_{1}(1-u_{1}\kappa)}\right\}\right.+\sum_{j\geq4} \left\{\left.1-N_{-}HRMSM_{j}^{\eta_{1,2}n_{1}(1-u_{1}\kappa)}\right\}\right.$ | (28) |
| --- | --- |
| $\sum_{j\geq4} \lambda_{3,j}^{1}(t)=\sum_{j\geq4} \left\{\left.1-N_{-}prepLRMSM_{j}^{\eta_{1,1}n_{1}(1-u_{1}\kappa)}\right\}\right.+\sum_{j\geq4} \left\{\left.1-N_{-}prepHRMSM_{j}^{\eta_{1,2}n_{1}(1-u_{1}\kappa)}\right\}\right.$ | (29) |

Transmission forces for HRMSM not in PrEP and in PrEP are:

| $\sum_{j\geq4} \lambda_{1,j}^{2}(t)=\sum_{j\geq4} \lambda_{2,j}^{2}(t)=\sum_{j\geq4} \left\{\left.1-N_{-}LRMSM_{j}^{\eta_{2,1}n_{2}(1-u_{2}\kappa)}\right\}\right.+\sum_{j\geq4} \left\{\left.1-N_{-}HRMSM_{j}^{\eta_{2,2}n_{2}(1-u_{2}\kappa)}\right\}\right.$ | (30) |
| --- | --- |
| $\sum_{j\geq4} \lambda_{3,j}^{2}(t)=\sum_{j\geq4} \left\{\left.1-N_{-}prepLRMSM_{j}^{\eta_{2,1}n_{2}(1-u_{2}\kappa)}\right\}\right.+\sum_{j\geq4} \left\{\left.1-N_{-}prepHRMSM_{j}^{\eta_{2,2}n_{2}(1-u_{2}\kappa)}\right\}\right.$ | (31) |

These forces of infection will vary dynamically as the proportion of the population with HIV changes, and form the core of the infectious process. The individual probabilities of infection defined in equations (24) – (27) are primarily dependent upon assumptions about the number of sexual partners and the impact of identification and treatment on partner numbers. They will also be significantly changed by ART, which affects the value of $\sigma$ directly. Equations (28) – (31) are further affected by assumptions about condom use in each group, and by the assortativity assumptions made in equations (18) – (21).

## Intervention types and assumptions

We projected a base-case model that assumed current Chinese HIV treatment guidelines were followed for the next 20 years with no change in testing uptake and treatment entry rates. Against this base case we modeled several alternative scenarios and all of their combinations:

1. Scenario 1: Test-and-treat strategy that is fully compliant with the WHO 90-90-90 recommendations, representing the current HIV treatment guidelines implemented at much greater effectiveness than the current standard. This scenario assumed annual testing rates of 90% for all MSM, with an ART utilization rate of 90% for all diagnosed PLWH, and 90% of all PLWH receiving ART achieving viral suppression (i.e., 90% ART effectiveness).
2. Scenarios 2 – 4: PrEP for high-risk MSM with coverage of 25%, 50% or 75% of the entire population of identified HIV-negative high-risk MSM. This case assumed that PrEP is provided to 25%, 50%, or 75% of the identified susceptible high-risk MSM, with 60% PrEP effectiveness. The testing rate remained at the current level of 37%.
3. Scenarios 5 – 7: PrEP for high-risk MSM (with the same three levels of PrEP coverage) combined with expanded annual voluntary counseling and testing (VCT) of 90% MSM. In this case, PrEP is provided to 25%, 50%, or 75% of the identified susceptible high-risk MSM, with expansion of the VCT. This scenario assumed expanded annual testing rates of 90% for all MSM, with 60% PrEP effectiveness. As VCT is expanded, more and more susceptible MSM enter PrEP once they are identified as HIV-negative.
4. Scenarios 8 – 10: PrEP for high-risk MSM (with the same three levels of PrEP coverage) combined with the test-and-treat strategy. These strategies included all three biomedical interventions (i.e., ART, VCT and PrEP). This scenario assumed annual testing rates of 90% for all MSM, with an ART utilization rate of 90% for all diagnosed PLWH, and 90% of all PLWH receiving ART achieving viral suppression. Again, PrEP is provided to 25%, 50%, or 75% of the identified susceptible high-risk MSM, with 60% PrEP effectiveness.

## Model outputs

In order to capture the epidemiological trends in HIV over the future forecast range, we calculated HIV prevalence, the HIV incidence rate, and cumulative HIV infections. For economic outcomes, we calculated QALYs.

### Epidemiological Outcomes

| HIV prevalence in risk-group *i* is given by, $P_{i}=\sum_{\text{j=4}}^{15} X_{i,j}(t)/\sum_{\forall j} X_{i,j}(t)$ | (32) |
| --- | --- |
| New infections in risk-group *i*, $NI_{i}=\sum_{j\geq4} \lambda_{1,j}^{i}(t)\times X_{i,1}(t)+\sum_{j\geq4} \lambda_{2,j}^{i}(t)\times X_{i,2}(t)+\sum_{j\geq4} \lambda_{3,j}^{i}(t)\times X_{i,3}(t);$ | (33) |
| Cumulative new infections in risk-group *i,* $CI_{i}=\int_{0}^{\text{t}} \left(\sum_{j\geq4} \lambda_{1,j}^{i}(t)\times X_{i,1}(t)+\sum_{j\geq4} \lambda_{2,j}^{i}(t)\times X_{i,2}(t)+\sum_{j\geq4} \lambda_{3,j}^{i}(t)\times X_{i,3}(t)\right)dt$ | (34) |

### Economic Outcomes

We measured economic outcomes as QALYs, which were in turn based on cost calculations for the different interventions. These are calculated in equations (35) to (40).

QALYs in risk-group *i* are defined using an integral expression to incorporate the discount rate, *r*, as follows:

| $\text{Q}^{\text{i}}=\int_{0}^{20} e^{-rt}\sum_{\forall j} q_{j}^{i}X_{i,j}(t)dt$ | (35) |
| --- | --- |

Where here $q_{j}^{i}$ measures the quality of life adjustment for population compartment j in risk group *i* ($q_{j}^{i}\in[0,1])$.

Total QALYs are then defined as:

| $\text{TQ}=\sum_{\forall\text{i}} \text{Q}^{\text{i}}$ | (36) |
| --- | --- |
| The cost of ART in risk-group *i* is also discounted: $\text{C}_{\text{ART}}^{i}=\int_{0}^{20} e^{-rt}\sum_{\forall j} c_{j}^{i}X_{i,j}(t)dt$ | (37) |
| As is the cost of VCT in risk-group *i*: $\text{C}_{\text{screen}}^{i}=\int_{0}^{20} e^{-rt}\left[\left(c_{neg}\psi_{1}^{i}X_{i,1}(t)\right)+\sum_{j=4,7,10,13} c_{pos}\psi_{j}^{i}X_{i,j}(t)\right]dt$ | (38) |
| The Cost for PrEP is defined in terms of the size only of the single PrEP group, and is discounted in the same way: $\text{C}_{PrEP}=\int_{0}^{20} e^{-rt}c_{PrEP}X_{i,3}(t)dt$ | (39) |
| Finally, health care cost in risk-group *i* is given by: $\text{C}_{\text{HC}}^{i}=\int_{0}^{20} e^{-rt}\sum_{\forall j} c'_{j}^{i}X_{i,j}(t)dt$ | (40) |

We calculated QALYs over the 20 year period from 2017 – 2037, with an annual discount rate of 3%.

## Model simulation

The model equations were used to generate estimates of the number of people with HIV and the population size in every compartment of the model for each risk group for every year from 2005 – 2037. The model projection period began in 2017, and data from 2005 – 2015 were used for calibration. This means that the estimated rate of change of each box at each point in time was calculated from equations (1) – (15) for each group and used to update the value of all the population numbers at the next time point. A time step of 1 month was used. The full heuristic for updating from step t to step t+1 is as follows:

1. Identify population at time t for all 15 compartments and both risk groups, $X_{i,j}(t)$, and obtain values of all parameters for compartment i, risk group j, from Table S2 (the values of $X_{\left\{ i,j \right\}}(0)$ are given in Table S3);
2. Calculate the forces of infection at time t from equations (22) – (31), which identify the total numbers of partners in each group, probability of an infectious contact, and total force of infection
3. Calculate the instantaneous rate of change of all population groups, $\frac{dX_{\left\{ i,j \right\}}}{dx}(t)$ using equations (1) – (15)
4. Calculate the value of population in all population and risk groups at time t+1 with a simple update, $X\left( t+1 \right)=X\left( t \right)+\frac{dX_{\left\{ i,j \right\}}}{dx}(t)$

The update stage (4) assumes that all rates have been adjusted from annual numbers to monthly numbers using standard formulae.^38^ Final estimates are aggregated to yearly values to ensure consistency with observed numbers, either as a sum of monthly values (incidence) or as mid-year averages (prevalence). Note that incidence is obtained from some components of equations (4) and (5). The equation for calculation of incidence is shown in equation (33).

## Model calibration

We calibrated the model by comparing projected prevalence values for the period 2005 – 2015 against observed prevalence estimates for these years, obtained from previously published national data^7,8^. These observed estimates are based on Chinese surveillance system^8^ and systematic review^7^.

For each year the projected value was compared with the observed prevalence estimate using the deviance formula, which is:

$$D_{i}=2Y_{i}\ln\left( \frac{Y_{i}}{\mu_{i}} \right)-(Y_{i}-\mu_{i})$$

for year *i*. Thus for the entire period 2005-2015, with 10 years of calibration data, the deviance formula becomes

$D=2\sum_{i} Y_{i}\ln\left( \frac{Y_{i}}{\mu_{i}} \right)-(Y_{i}-\mu_{i})$ (41)

where $i\in[2005,2015]$. Of course the factor of 2 is irrelevant when comparing between deviances. For two models A and B, the modeled projection A over the 2005 – 2015 period is considered better than the modeled projection B if $D_{A}<D_{B}$ and the goal of parameter selection is to find the model from among a large set of candidate models which minimizes D.

Based on this deviance statistic we then developed a model selection process in which we randomly sampled from key parameters simultaneously, generated predicted prevalences for the period 2005 – 2015 and estimated the deviance. This was performed for 1000 random samples of the parameter sets, and the 400 models with the lowest deviance values were retained as final sensitivity estimates. The weighted mean of these 400 models was used to project the base case for all results in the main text, and akk 400 models were used to establish uncertainty ranges for all values.

Parameter estimates were sampled using Beta distributions, with the parameters of the Beta distribution set to ensure that there was a tight peak around the central parameter value assigned in Table S2. Table S4 shows the specific Beta distributions used for each parameter that was included in sensitivity analysis.

Table S4 Distribution and ranges of key parameters for sensitivity analysis

| Variables | Value | Range | Distribution |
| --- | --- | --- | --- |
| Condom use with same-sex partners, % | 36.3% | 27.2%–45.4% | [0.5*Beta(2,2)+0.75]*36.3% |
| Average annual partner number of MSM | 5 | 4–6 | 2*Beta(2,2)+4 |
| Average annual partner number of high-risk MSM | 15 | 12.5–17.5 | 5*Beta(2,2)+12.5 |
| Mixing parameter | 0.3 | 0.15–0.45 | 0.3*Beta(2,2)+0.15 |
| Annual HIV-related health care cost, Int.$ |  |  |  |
| HIV positive (CD4>500) | 2,624 | 1,968–3280 | [0.5*Beta(2,2)+0.75]*2624 |
| HIV positive (200≤CD4≤500) | 4,406 | 3,305–5,508 | [0.5*Beta(2,2)+0.75]*4406 |
| AIDS (CD4<200) | 13,585 | 10,189–16,981 | [0.5*Beta(2,2)+0.75]*13585 |
| HIV positive (CD4>500) with ART | 2,624 | 1,968–3,280 | [0.5*Beta(2,2)+0.75]*2624 |
| HIV positive (200≤CD4≤500) with ART | 3,928 | 2,946–4,910 | [0.5*Beta(2,2)+0.75]*3928 |
| AIDS (CD4<200) with ART | 6,182 | 4,637–7,728 | [0.5*Beta(2,2)+0.75]*6182 |
| Annual non-HIV related health care cost, Int.$ | 764 | 573–955 | [0.5*Beta(2,2)+0.75]*764 |
| Annual cost of PrEP, Int.$ | 6,909 | 5,182–8,636 | [0.5*Beta(2,2)+0.75]*6909 |
| Annual cost of ART, Int.$ | 6,540 | 4,905–8,175 | [0.5*Beta(2,2)+0.75]*6540 |

# Supplementary results

## Sensitivity analysis

The projection of the HIV epidemic among MSM in China influenced by the effects of the ten individual and combined interventions is presented in Figure S2. The sensitivity ranges of the epidemiological impact of the ten interventions on HIV prevalence and HIV incidence rates are also plotted in Figure S2.

### PrEP effectiveness

In addition to the uncertainty analysis, we also tested the robustness of our findings to changes in the assumption about PrEP effectiveness, which depends on PrEP adherence (Figure S3 and Table S5). With a decrease in PrEP effectiveness from 60% to 30%, under the PrEP 25%/50%/75% high-risk MSM strategies (scenarios 2 – 4), the total number of new infections prevented will drop from 0.09 – 0.20 million to 0.05 – 0.12 million (12.1 – 25.7% *vs.* 7.0 – 14.9%, Figure S3 and Table S5); the cost per QALY gained will increase from 17277 – 18452 Int.$ to 29902 – 30414 Int.$ (Table S5). With this assumption of poor PrEP adherence, even the joint strategy of test-and-treat and PrEP 75% high-risk MSM will not achieve HIV elimination by the end of 2037.

If instead of 60% effectiveness, PrEP increases to 90% effectiveness, under scenario 2 – 4, the total number of new infection prevented will increase to 0.14 – 0.29 million (18.4 – 37.8%, Figure S3 and Table S5); furthermore, the cost of PrEP 25%/50%/75% high-risk MSM strategies will decrease to 11679 – 13280 Int.$/QALY (Table S5).

### Risk compensation

In the analysis of risk compensation, it is assumed that all PrEP users in scenario 2 – 10 completely stopped using condoms with their sex partners. With an assumed ideal PrEP effectiveness of 90%, the total number of new infections prevented and HIV epidemic situation were not sensitive to sexual risk compensation (Table S6 (a)).

If instead of 90% effectiveness, PrEP reduces to 60% effectiveness (Table S6 (b)), over 20 years, the PrEP 25%/50%/75% high-risk MSM strategies without risk compensation and with risk compensation will prevent 0.09 – 0.20 million (12.1 – 25.7%) and 0.07 – 0.14 million (8.6 – 18.3%) new infections, respectively.

With a decrease in PrEP effectiveness from 90% to 30% (Table S6 (c)), the number of infections prevented will reduce by around 70% in scenarios 2 – 4 (i.e., PrEP 25%/50%/75% high-risk MSM) with risk compensation compared to the scenarios without risk compensation (Table S6 (c) in the supplement), suggesting that risk compensation could be a significant hindrance to the impact of this strategy in situations where PrEP effectiveness is low.

Figure S2 The effects of the ten interventions on HIV prevalence and HIV incidence rates with sensitivity range over 2005~2037, with PrEP effectiveness of 60%.

| 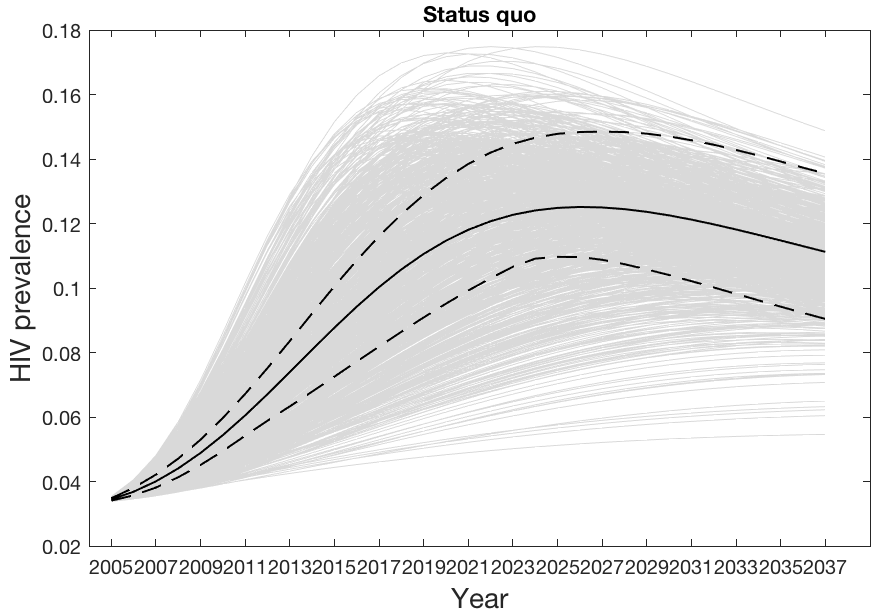 | 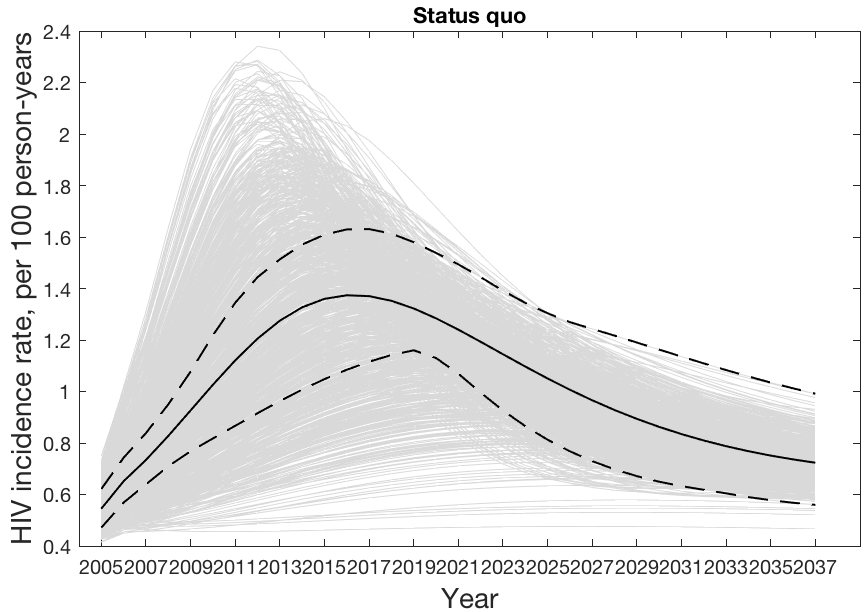 |
| --- | --- |
| 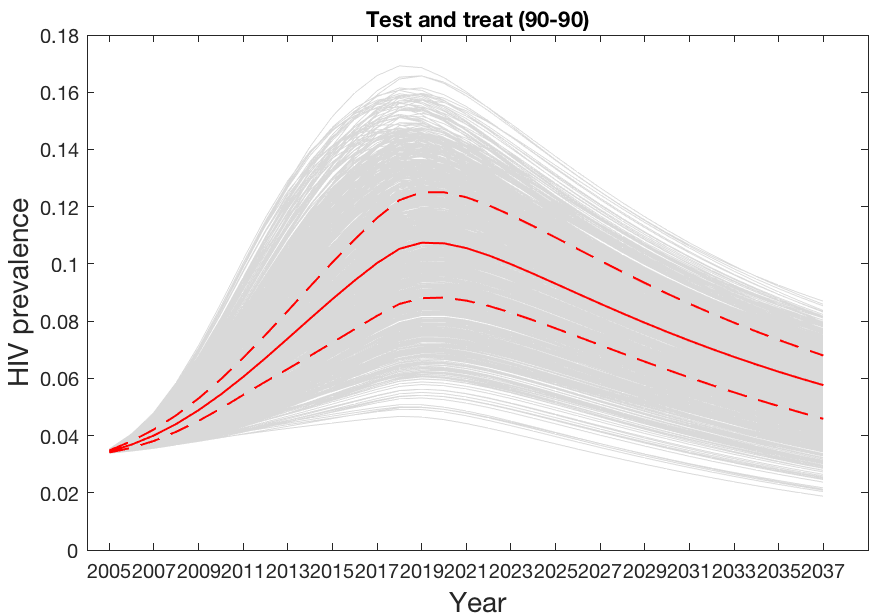 | 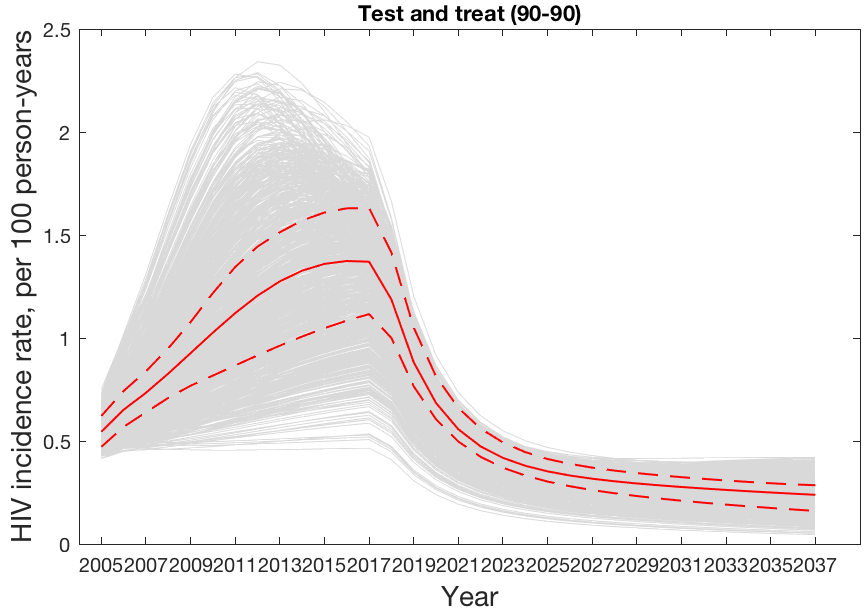 |
| 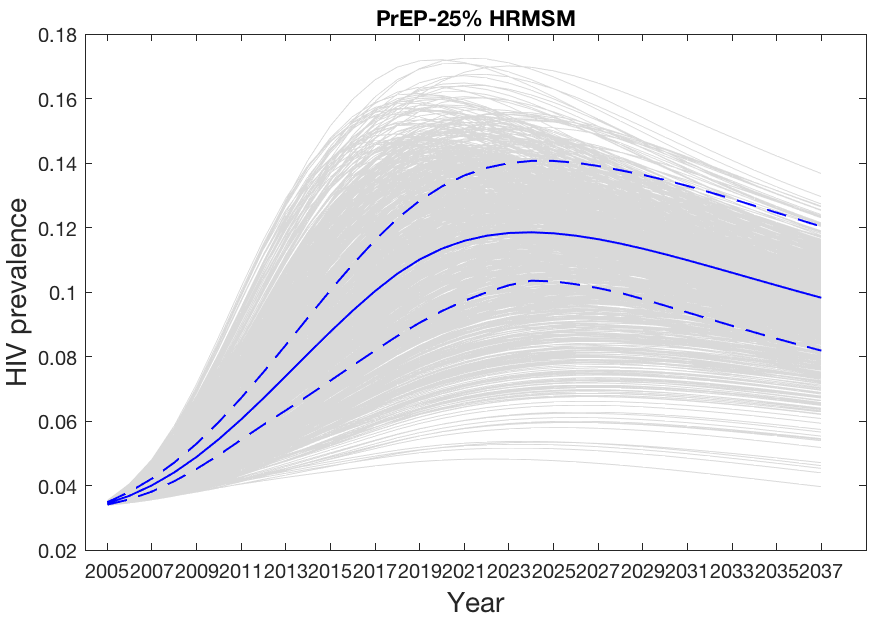 | 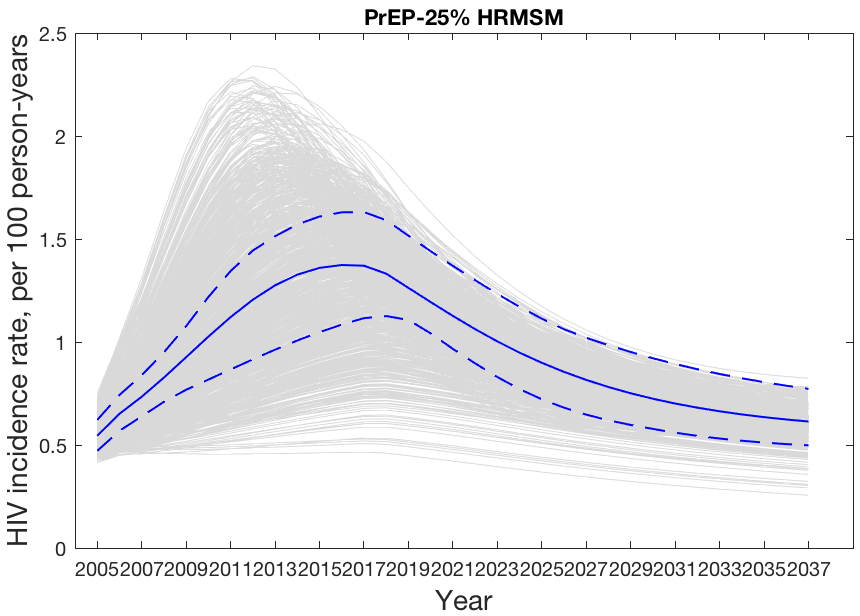 |
| 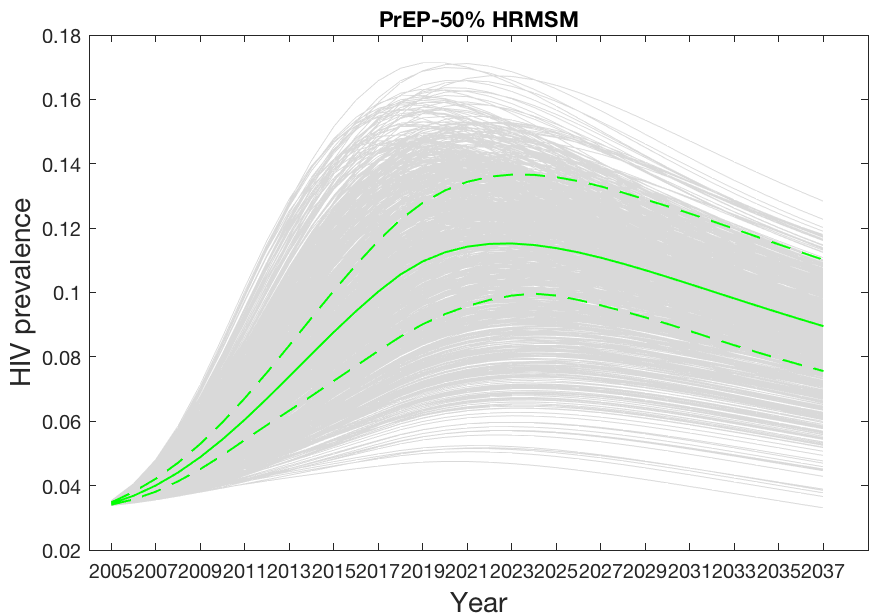 | 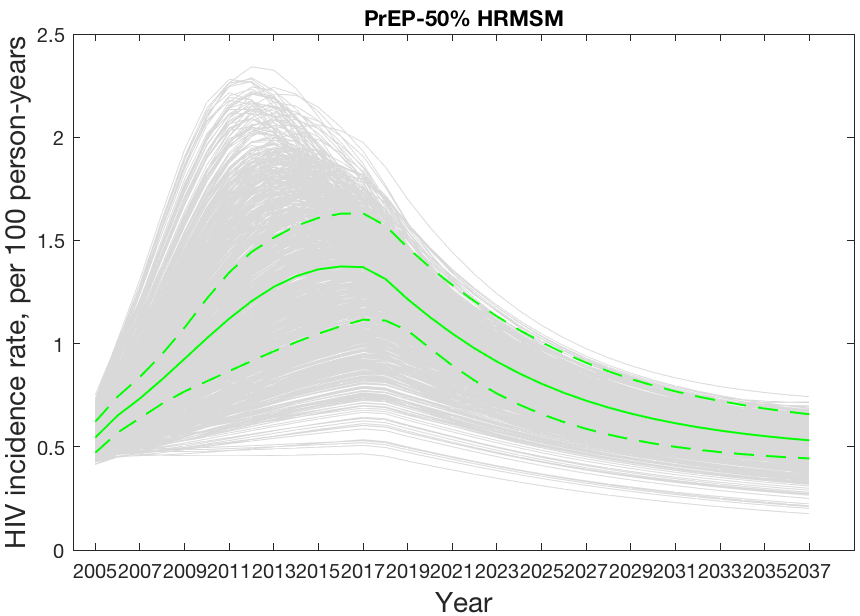 |
| 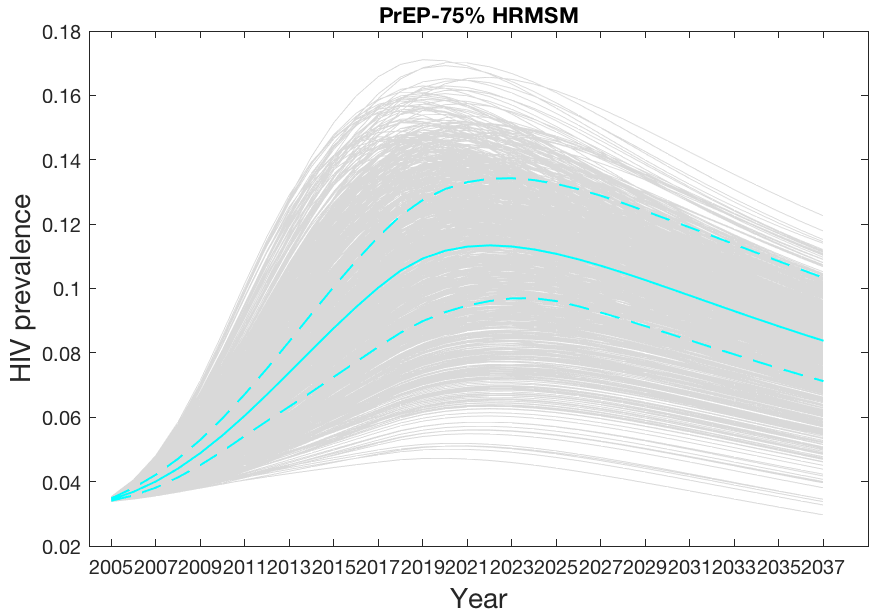 | 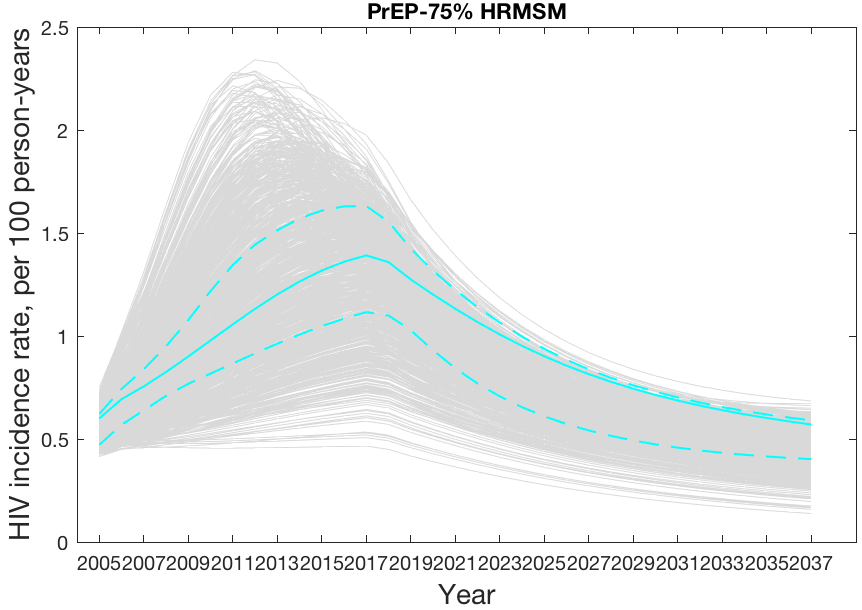 |
| 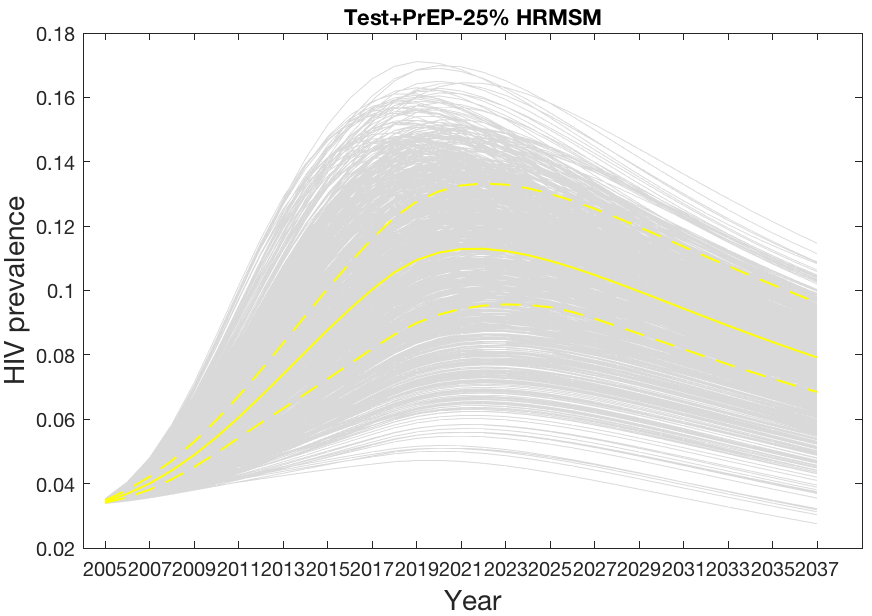 | 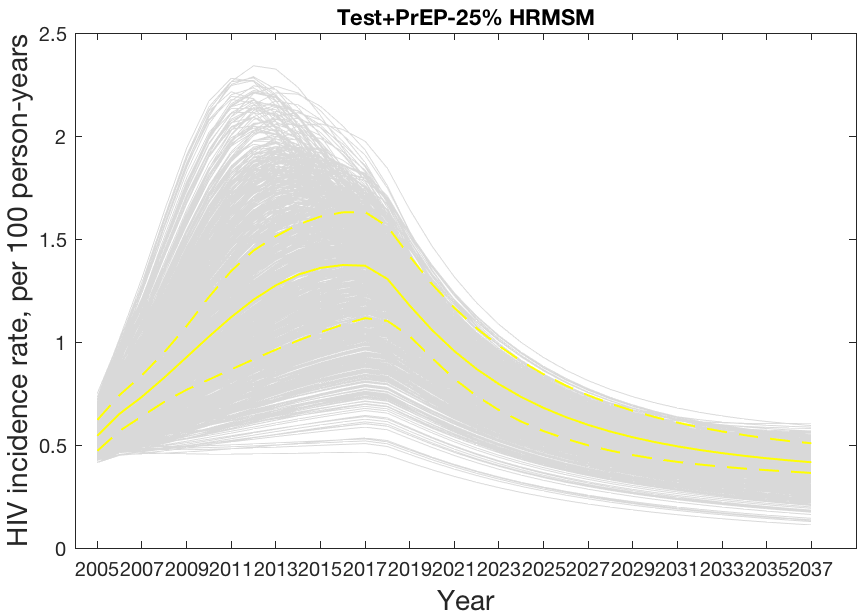 |
| 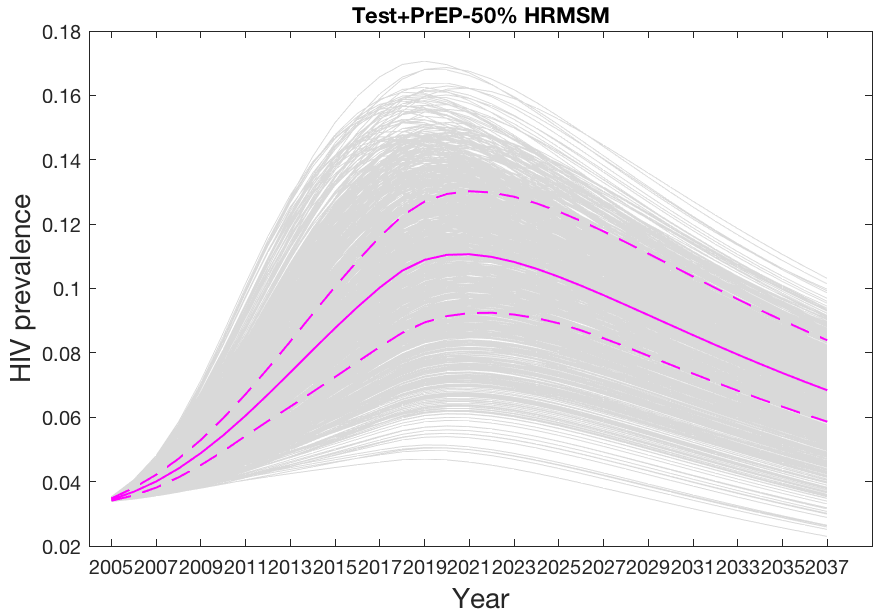 | 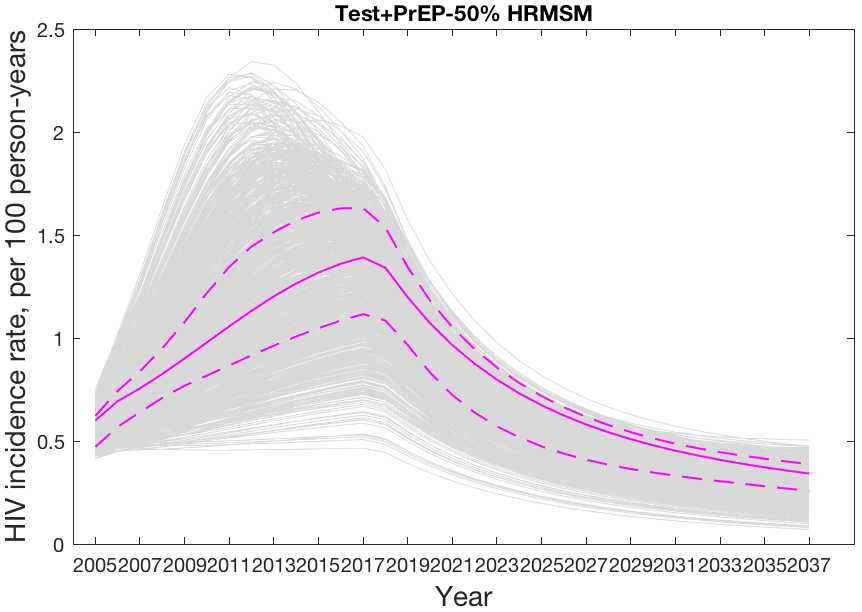 |
| 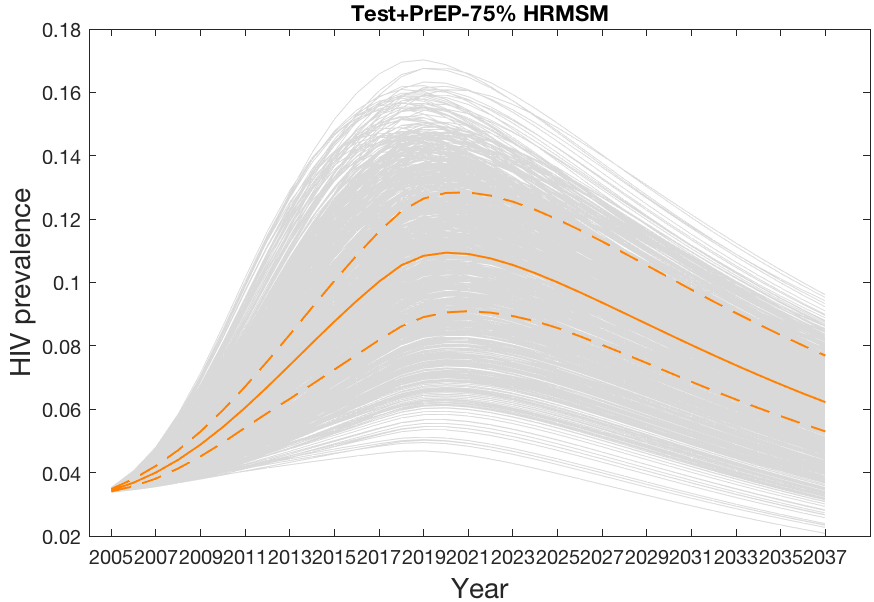 | 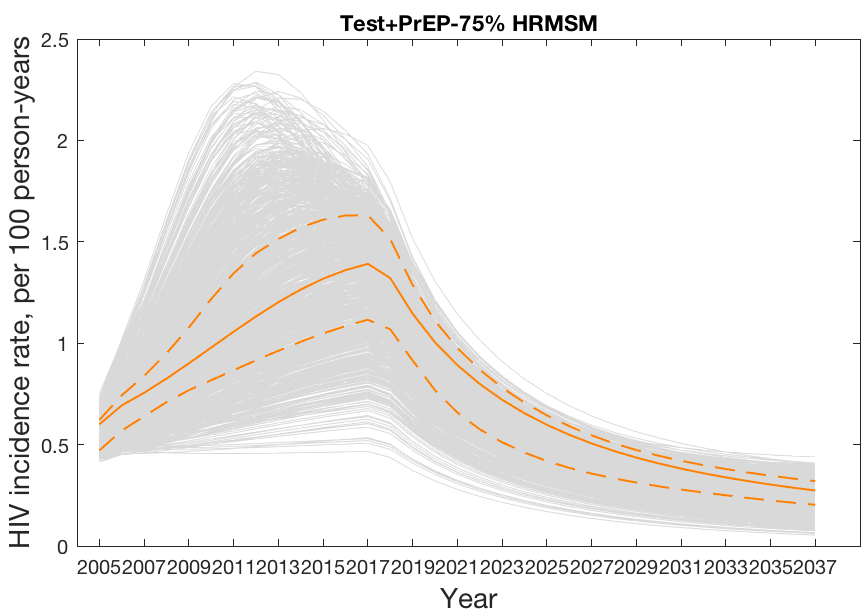 |
| 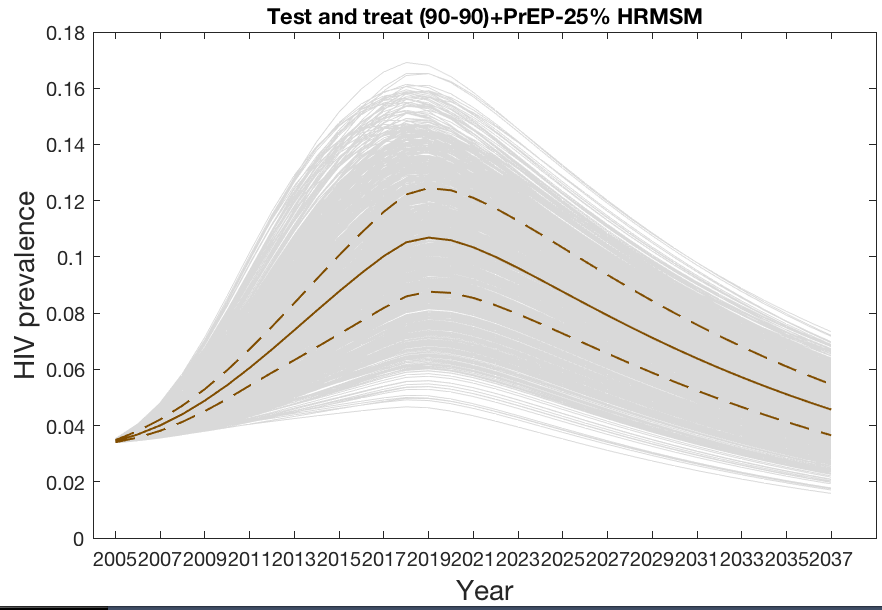 | 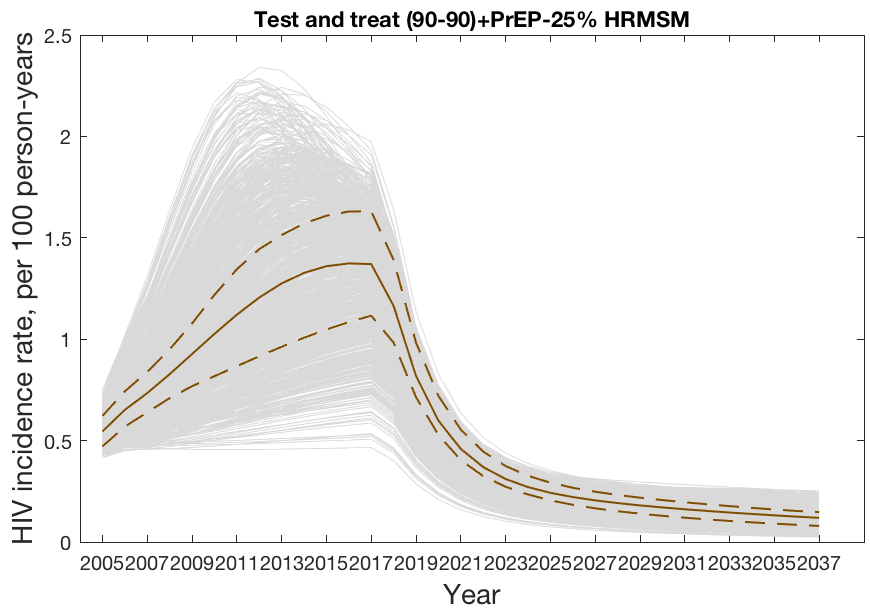 |
| 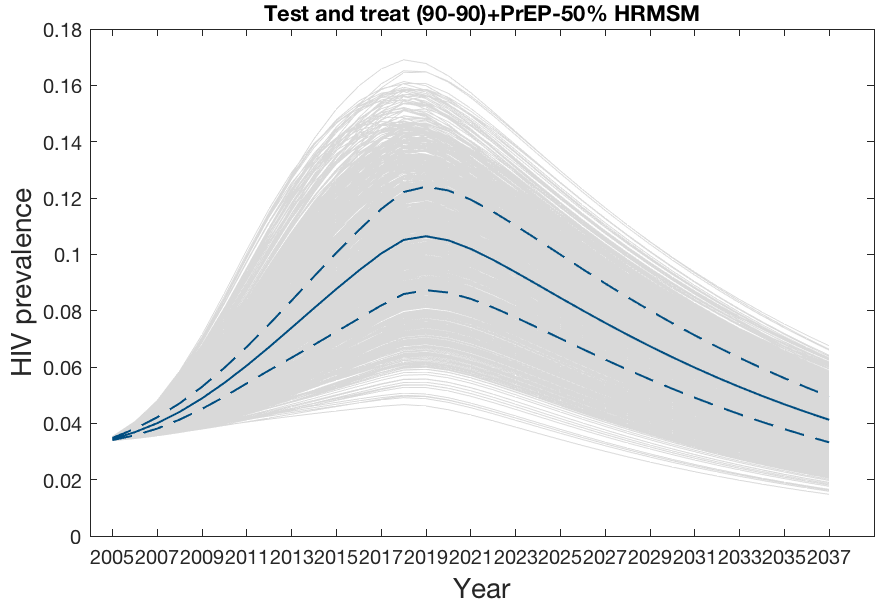 | 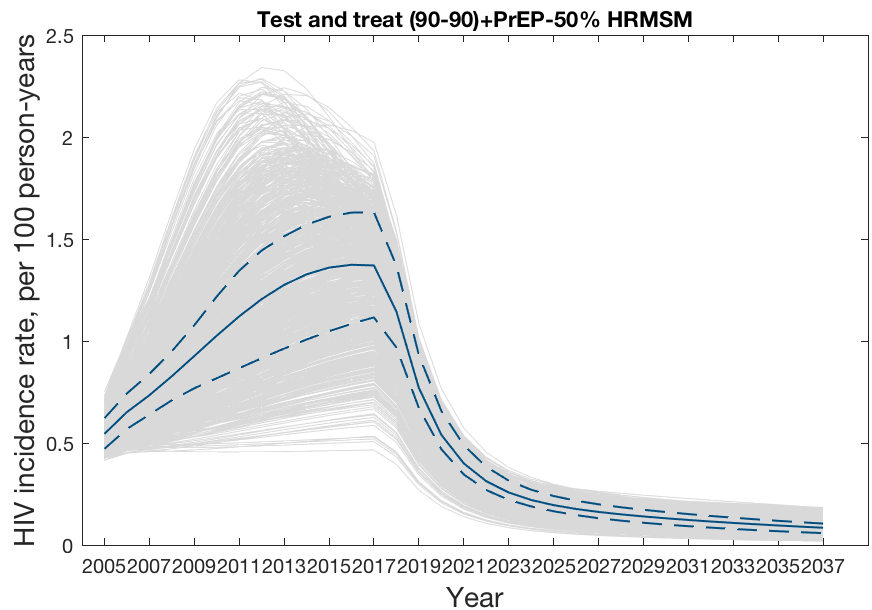 |
| 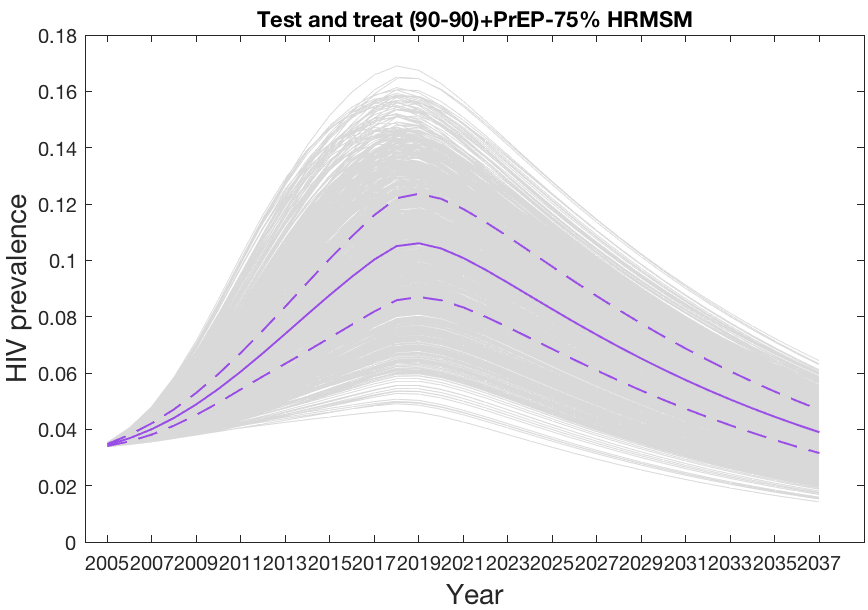 | 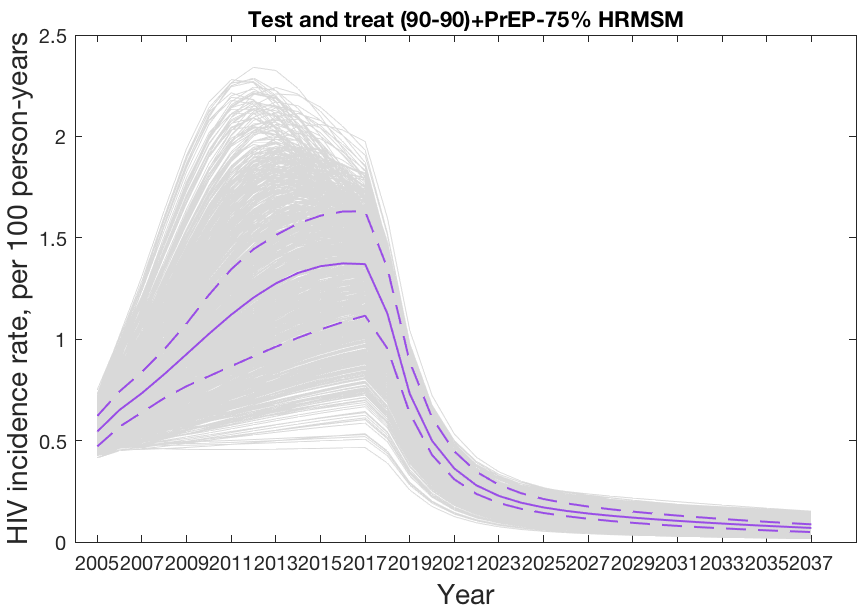 |

Solid line: weighted mean;

Dotted line: the upper and lower bound of the sensitivity range;

Figure S3 Percentage of HIV infections prevented under all interventions with various PrEP effectiveness

S2: PrEP 25% high-risk MSM;

S3: PrEP 50% high-risk MSM;

S4: PrEP 75% high-risk MSM;

S5: Test and PrEP 25% high-risk MSM;

S6: Test and PrEP 50% high-risk MSM;

S7: Test and PrEP 75% high-risk MSM;

S8: Test and treat (90-90) and PrEP 25% high-risk MSM;

S9: Test and treat (90-90) and PrEP 50% high-risk MSM;

S10: Test and treat (90-90) and PrEP 75% high-risk MSM.

Table S5 Sensitivity Analysis on Cost-effectiveness of Varying Strategy by PrEP Effectiveness

(a) With PrEP effectiveness of 90%

|  | Total infection over 20 years (2018~2037), million | HIV infections prevented over 20 years, million | HIV infections prevented over 20 years, % | HIV prevalence at 2037, % | HIV incidence rate at 2037, per 100 person-years | Incremental costs, Int.$ (billion) | Incremental QALYs, million | Average CER, Int.$/QALY, relative to base case | Saving in healthcare cost, Int.$ (billion) |
| --- | --- | --- | --- | --- | --- | --- | --- | --- | --- |
| The baseline case | 0.78 (0.60–1.00) | - | - | 11.2 (9.1–14.2) | 0.73 (0.56–0.98) | - | - | - | - |
| Test and treat (90-90) | 0.33 (0.28–0.39) | 0.45 (0.28–0.62) | 57.9% (46.4%–66.5%) | 5.8 (4.8–6.8) | 0.24 (0.18–0.29) | 2.13 (0.61–4.03) | 1.22 (0.94–1.53) | 1,754 (462–3,960) | 8.86 (5.33–13.92) |
| PrEP-25% HRMSM | 0.64 (0.52–0.80) | 0.14 (0.08–0.20) | 18.4% (13.7%–23.7%) | 9.2 (8.0–11.7) | 0.55 (0.47–0.71) | 3.08 (1.77–5.17) | 0.26 (0.18–0.33) | 11,679 (8,009–16,245) | 2.59 (1.47–4.14) |
| PrEP-50% HRMSM | 0.54 (0.45–0.69) | 0.24 (0.14–0.32) | 30.2% (23.7%–37.3%) | 7.9 (6.8–10.1) | 0.43 (0.36–0.55) | 5.46 (3.17–9.04) | 0.43 (0.30–0.53) | 12,627 (8,624–17,830) | 4.23 (2.48–6.60) |
| PrEP-75% HRMSM | 0.48 (0.41–0.62) | 0.29 (0.19–0.40) | 37.8% (30.7%–45.3%) | 7.1 (6.1–9.1) | 0.34 (0.29–0.45) | 7.23 (4.24–11.84) | 0.54 (0.38–0.66) | 13,280 (9,037–18,903) | 5.29 (3.18–8.12) |
| Test+PrEP-25% HRMSM | 0.47 (0.40–0.59) | 0.31 (0.19–0.43) | 40.2% (32.3%–48.0%) | 6.9 (6.0–8.7) | 0.31 (0.26–0.39) | 6.90 (4.52–10.50) | 0.62 (0.43–0.77) | 11,199 (7,877–15,224) | 5.61 (3.32–8.84) |
| Test+PrEP-50% HRMSM | 0.36 (0.31–0.47) | 0.42 (0.28–0.55) | 53.4% (46.1%–60.5%) | 5.6 (4.7–7.0) | 0.18 (0.14–0.24) | 10.96 (7.00–16.84) | 0.81 (0.59–0.98) | 13,525 (9,424–18,860) | 7.48 (4.66–11.35) |
| Test+PrEP-75% HRMSM | 0.31 (0.26–0.40) | 0.47 (0.33–0.61) | 60.4% (54.1%–66.7%) | 4.9 (4.0–6.2) | 0.13 (0.10–0.17) | 13.77 (8.80–21.08) | 0.93 (0.70–1.11) | 14,863 (10,294–20,988) | 8.56 (5.47–12.74) |
| Test and treat (90-90)+PrEP 25% HRMSM | 0.22 (0.19–0.27) | 0.56 (0.39–0.73) | 71.7% (64.2%–77.3%) | 4.3 (3.5–5.1) | 0.09 (0.07–0.12) | 9.93 (7.54–13.17) | 1.39 (1.10–1.71) | 7,163 (5,036–9,806) | 10.68 (6.89–16.08) |
| Test and treat (90-90)+PrEP 50% HRMSM | 0.18 (0.15–0.23) | 0.60 (0.43–0.77) | 76.8% (71.1%–81.3%) | 3.8 (3.1–4.6) | 0.06 (0.04–0.08) | 14.59 (10.55–20.23) | 1.47 (1.18–1.80) | 9,925 (6,914–13,896) | 11.49 (7.59–17.02) |
| Test and treat (90-90)+PrEP 75% HRMSM | 0.16 (0.13–0.20) | 0.62 (0.45–0.79) | 79.5% (74.9%–83.4%) | 3.5 (2.9–4.3) | 0.05 (0.03–0.06) | 17.59 (12.50–24.69) | 1.52 (1.23–1.86) | 11,571 (8,042–16,312) | 11.96 (7.99–17.57) |

(b) With PrEP effectiveness of 60%

|  | Total infection over 20 years (2018~2037), million | HIV infections prevented over 20 years, million | HIV infections prevented over 20 years, % | HIV prevalence at 2037, % | HIV incidence rate at 2037, per 100 person-years | Incremental costs, Int.$ (billion) | Incremental QALYs, million | Average CER, Int.$/QALY, relative to base case | Saving in healthcare cost, Int.$ (billion) |
| --- | --- | --- | --- | --- | --- | --- | --- | --- | --- |
| The baseline case | 0.78 (0.60–1.00) | - | - | 11.2 (9.1–14.2) | 0.73 (0.56–0.98) | - | - | - | - |
| Test and treat (90-90) | 0.33 (0.28–0.39) | 0.45 (0.28–0.62) | 57.9% (46.4%–66.5%) | 5.8 (4.8–6.8) | 0.24 (0.18–0.29) | 2.13 (0.61–4.03) | 1.22 (0.94–1.53) | 1,754 (462–3,960) | 8.86 (5.33–13.92) |
| PrEP-25% HRMSM | 0.68 (0.57–0.87) | 0.09 (0.06–0.14) | 12.1% (8.7%–16.7%) | 9.8 (8.2–12.8) | 0.61 (0.50–0.80) | 3.08 (1.76–5.30) | 0.18 (0.12–0.23) | 17,277 (12,127–23,711) | 1.73 (0.94–2.79) |
| PrEP-50% HRMSM | 0.62 (0.52–0.80) | 0.16 (0.10–0.23) | 20.2% (15.1%–26.9%) | 8.9 (7.5–11.7) | 0.53 (0.44–0.70) | 5.34 (3.07–9.10) | 0.30 (0.21–0.38) | 17,979 (12,631–24,991) | 2.87 (1.60–4.52) |
| PrEP-75% HRMSM | 0.57 (0.49–0.75) | 0.20 (0.13–0.29) | 25.7% (19.6%–33.3%) | 8.4 (7.0–11.0) | 0.47 (0.40–0.63) | 6.98 (4.05–11.80) | 0.38 (0.27–0.48) | 18,452 (12,972–25,846) | 3.64 (2.06–5.64) |
| Test+PrEP-25% HRMSM | 0.53 (0.46–0.68) | 0.24 (0.15–0.34) | 30.8% (23.9%–39.3%) | 7.9 (6.8–10.2) | 0.42 (0.36–0.53) | 6.83 (4.39–10.62) | 0.49 (0.37–0.61) | 13,835 (9,600–18,598) | 4.36 (2.46–6.82) |
| Test+PrEP-50% HRMSM | 0.46 (0.39–0.59) | 0.32 (0.21–0.44) | 41.0% (33.3%–49.9%) | 6.8 (5.8–8.8) | 0.31 (0.26–0.40) | 10.61 (6.65–16.76) | 0.64 (0.48–0.78) | 16,636 (11,595–22,919) | 5.77 (3.37–8.86) |
| Test+PrEP-75% HRMSM | 0.41 (0.35–0.53) | 0.36 (0.25–0.49) | 47.0% (39.3%–55.6%) | 6.2 (5.2–8.1) | 0.25 (0.20–0.33) | 13.20 (8.25–20.85) | 0.73 (0.56–0.88) | 18,110 (12,660–25,289) | 6.64 (3.95–10.12) |
| Test and treat (90-90)+PrEP 25% HRMSM | 0.24 (0.21–0.30) | 0.53 (0.39–0.68) | 68.3% (61.1%–75.4%) | 4.6 (3.7–5.6) | 0.12 (0.08–0.15) | 10.11 (7.42–13.49) | 1.33 (1.12–1.69) | 7,574 (5,166–10,243) | 10.17 (6.30–16.33) |
| Test and treat (90-90)+PrEP 50% HRMSM | 0.21 (0.18–0.26) | 0.56 (0.43–0.72) | 72.8% (66.6%–78.7%) | 4.1 (3.4–5.1) | 0.09 (0.06–0.11) | 14.69 (10.27–20.60) | 1.40 (1.19–1.76) | 10,485 (7,199–14,484) | 10.83 (6.82–17.25) |
| Test and treat (90-90)+PrEP 75% HRMSM | 0.19 (0.16–0.24) | 0.58 (0.45–0.74) | 75.2% (69.7%–80.5%) | 3.9 (3.2–4.8) | 0.07 (0.05–0.09) | 17.63 (12.14–25.06) | 1.44 (1.23–1.81) | 12,218 (8,415–16,992) | 11.23 (7.13–17.81) |

(c) With PrEP effectiveness of 30%

|  | Total infection over 20 years (2018~2037), million | HIV infections prevented over 20 years, million | HIV infections prevented over 20 years, % | HIV prevalence at 2037, % | HIV incidence rate at 2037, per 100 person-years | Incremental costs, Int.$ (billion) | Incremental QALYs, million | Average CER, Int.$/QALY, relative to base case | Saving in healthcare cost, Int.$ (billion) |
| --- | --- | --- | --- | --- | --- | --- | --- | --- | --- |
| The baseline case | 0.78 (0.60–1.00) | - | - | 11.2 (9.1–14.2) | 0.73 (0.56–0.98) | - | - | - | - |
| Test and treat (90-90) | 0.33 (0.28–0.39) | 0.45 (0.28–0.62) | 57.9% (46.4%–66.5%) | 5.8 (4.8–6.8) | 0.24 (0.18–0.29) | 2.13 (0.61–4.03) | 1.22 (0.94–1.53) | 1,754 (462–3,960) | 8.86 (5.33–13.92) |
| PrEP-25% HRMSM | 0.72 (0.59–0.91) | 0.05 (0.03–0.09) | 7.0% (4.7%–10.2%) | 10.4 (8.6–13.2) | 0.66 (0.52–0.86) | 3.17 (1.68–4.99) | 0.11 (0.07–0.15) | 29,902 (21,292–39,683) | 1.02 (0.51–1.61) |
| PrEP-50% HRMSM | 0.69 (0.57–0.86) | 0.09 (0.05–0.15) | 11.7% (8.1%–16.7%) | 9.9 (8.2–12.5) | 0.62 (0.50–0.80) | 5.38 (2.87–8.42) | 0.18 (0.12–0.24) | 30,222 (21,492–39,985) | 1.70 (0.87–2.65) |
| PrEP-75% HRMSM | 0.66 (0.55–0.83) | 0.12 (0.07–0.19) | 14.9% (10.4%–20.9%) | 9.6 (8.0–12.1) | 0.59 (0.48–0.76) | 6.94 (3.72–10.82) | 0.23 (0.15–0.30) | 30,414 (21,613–40,170) | 2.17 (1.12–3.35) |
| Test+PrEP-25% HRMSM | 0.60 (0.52–0.74) | 0.17 (0.10–0.28) | 22.3% (16.0%–30.6%) | 8.9 (7.7–11.0) | 0.52 (0.44–0.64) | 6.91 (4.20–10.05) | 0.39 (0.27–0.50) | 17,869 (12,418–24,877) | 3.30 (1.74–5.30) |
| Test+PrEP-50% HRMSM | 0.56 (0.48–0.69) | 0.22 (0.13–0.34) | 28.3% (20.9%–37.4%) | 8.3 (7.2–10.2) | 0.45 (0.40–0.57) | 10.48 (6.17–15.52) | 0.47 (0.34–0.60) | 22,174 (15,398–30,261) | 4.14 (2.23–6.53) |
| Test+PrEP-75% HRMSM | 0.53 (0.46–0.65) | 0.25 (0.15–0.38) | 32.0% (24.1%–41.5%) | 7.9 (6.8–9.7) | 0.42 (0.37–0.52) | 12.87 (7.51–19.12) | 0.53 (0.38–0.66) | 24,316 (16,907–32,913) | 4.68 (2.55–7.32) |
| Test and treat (90-90)+PrEP 25% HRMSM | 0.27 (0.23–0.32) | 0.51 (0.36–0.67) | 65.3% (56.6%–73.7%) | 4.9 (4.1–5.8) | 0.15 (0.11–0.19) | 10.40 (7.45–13.44) | 1.30 (1.06–1.61) | 7,986 (5,549–11,393) | 9.91 (5.99–15.72) |
| Test and treat (90-90)+PrEP 50% HRMSM | 0.24 (0.21–0.29) | 0.53 (0.38–0.69) | 68.6% (60.8%–76.1%) | 4.6 (3.8–5.4) | 0.12 (0.09–0.15) | 15.04 (10.17–20.22) | 1.35 (1.11–1.67) | 11,141 (7,691–15,528) | 10.40 (6.35–16.37) |
| Test and treat (90-90)+PrEP 75% HRMSM | 0.23 (0.20–0.27) | 0.55 (0.40–0.71) | 70.5% (63.2%–77.5%) | 4.4 (3.6–5.2) | 0.10 (0.08–0.13) | 17.97 (11.93–24.47) | 1.38 (1.14–1.70) | 13,025 (8,979–18,001) | 10.69 (6.57–16.77) |

Table S6 Impact of risk compensation on the epidemiological effects of all interventions with various PrEP effectiveness

(a) With PrEP effectiveness of 90%

| PrEP effectiveness=0.9 | | | | | | | | |
| --- | --- | --- | --- | --- | --- | --- | --- | --- |
|  | HIV infections prevented over 20 years, million | | HIV infections prevented over 20 years, % | | HIV prevalence at 2037, % | | HIV incidence rate at 2037, per 100 person-years | |
| Interventions | No risk compensation | With risk compensation | No risk compensation | With risk compensation | No risk compensation | With risk compensation | No risk compensation | With risk compensation |
| The baseline case | - | - | - | - | 11.2 (9.1–14.2) | 11.2 (9.1–14.2) | 0.73 (0.56–0.98) | 0.73 (0.56–0.98) |
| PrEP-25% HRMSM | 0.14 (0.08–0.20) | 0.13 (0.08–0.20) | 18.4% (13.7%–23.7%) | 17.3% (12.5%–22.8%) | 9.2 (8.0–11.7) | 9.3 (8.1–11.8) | 0.55 (0.47–0.71) | 0.56 (0.48–0.72) |
| PrEP-50% HRMSM | 0.24 (0.14–0.32) | 0.22 (0.13–0.31) | 30.2% (23.7%–37.3%) | 28.6% (21.7%–35.9%) | 7.9 (6.8–10.1) | 8.1 (7.0–10.3) | 0.43 (0.36–0.55) | 0.44 (0.38–0.57) |
| PrEP-75% HRMSM | 0.29 (0.19–0.40) | 0.28 (0.17–0.38) | 37.8% (30.7%–45.3%) | 35.8% (28.2%–43.8%) | 7.1 (6.1–9.1) | 7.3 (6.3–9.3) | 0.34 (0.29–0.45) | 0.34 (0.31–0.47) |
| Test+PrEP-25% HRMSM | 0.31 (0.19–0.43) | 0.30 (0.18–0.42) | 40.2% (32.3%–48.0%) | 38.8% (30.3%–46.9%) | 6.9 (6.0–8.7) | 7.1 (6.2–8.9) | 0.31 (0.26–0.39) | 0.33 (0.28–0.41) |
| Test+PrEP-50% HRMSM | 0.42 (0.28–0.55) | 0.40 (0.26–0.54) | 53.4% (46.1%–60.5%) | 51.5% (43.3%–59.2%) | 5.6 (4.7–7.0) | 5.8 (4.9–7.2) | 0.18 (0.14–0.24) | 0.20 (0.16–0.26) |
| Test+PrEP-75% HRMSM | 0.47 (0.33–0.61) | 0.46 (0.31–0.59) | 60.4% (54.1%–66.7%) | 58.5% (51.1%–65.4%) | 4.9 (4.0–6.2) | 5.1 (4.2–6.4) | 0.13 (0.10–0.17) | 0.14 (0.11–0.19) |
| Test and treat (90-90)+PrEP 25% HRMSM | 0.56 (0.39–0.73) | 0.55 (0.38–0.73) | 71.7% (64.2%–77.3%) | 71.2% (63.3%–77.1%) | 4.3 (3.5–5.1) | 4.3 (3.5–5.2) | 0.09 (0.07–0.12) | 0.10 (0.07–0.12) |
| Test and treat (90-90)+PrEP 50% HRMSM | 0.60 (0.43–0.77) | 0.59 (0.42–0.77) | 76.8% (71.1%–81.3%) | 76.2% (70.1%–81.0%) | 3.8 (3.1–4.6) | 3.8 (3.1–4.6) | 0.06 (0.04–0.08) | 0.06 (0.05–0.08) |
| Test and treat (90-90)+PrEP 75% HRMSM | 0.62 (0.45–0.79) | 0.61 (0.44–0.79) | 79.5% (74.9%–83.4%) | 78.9% (73.7%–83.0%) | 3.5 (2.9–4.3) | 3.6 (2.9–4.3) | 0.05 (0.03–0.06) | 0.05 (0.04–0.06) |

(b) With PrEP effectiveness of 60%

| PrEP effectiveness=0.6 | | | | | | | | |
| --- | --- | --- | --- | --- | --- | --- | --- | --- |
| Interventions | HIV infections prevented over 20 years, million | | HIV infections prevented over 20 years, % | | HIV prevalence at 2037, % | | HIV incidence rate at 2037, per 100 person-years | |
|  | No risk compensation | With risk compensation | No risk compensation | With risk compensation | No risk compensation | With risk compensation | No risk compensation | With risk compensation |
| The baseline case | - | - | - | - | 11.2 (9.1–14.2) | 11.2 (9.1–14.2) | 0.73 (0.56–0.98) | 0.73 (0.56–0.98) |
| PrEP-25% HRMSM | 0.09 (0.06–0.14) | 0.07 (0.04–0.11) | 12.1% (8.7%–16.7%) | 8.6% (5.5%–13.2%) | 9.8 (8.2–12.8) | 10.2 (8.5–13.2) | 0.61 (0.50–0.80) | 0.64 (0.53–0.84) |
| PrEP-50% HRMSM | 0.16 (0.10–0.23) | 0.11 (0.06–0.19) | 20.2% (15.1%–26.9%) | 14.4% (9.3%–21.5%) | 8.9 (7.5–11.7) | 9.6 (8.2–12.3) | 0.53 (0.44–0.70) | 0.59 (0.50–0.76) |
| PrEP-75% HRMSM | 0.20 (0.13–0.29) | 0.14 (0.08–0.23) | 25.7% (19.6%–33.3%) | 18.3% (12.0%–26.8%) | 8.4 (7.0–11.0) | 9.2 (7.9–11.7) | 0.47 (0.40–0.63) | 0.47 (0.47–0.70) |
| Test+PrEP-25% HRMSM | 0.24 (0.15–0.34) | 0.19 (0.11–0.30) | 30.8% (23.9%–39.3%) | 25.0% (17.6%–34.5%) | 7.9 (6.8–10.2) | 8.6 (7.4–10.8) | 0.42 (0.36–0.53) | 0.48 (0.42–0.60) |
| Test+PrEP-50% HRMSM | 0.32 (0.21–0.44) | 0.25 (0.15–0.38) | 41.0% (33.3%–49.9%) | 32.5% (23.2%–43.4%) | 6.8 (5.8–8.8) | 7.8 (6.7–9.7) | 0.31 (0.26–0.40) | 0.40 (0.34–0.49) |
| Test+PrEP-75% HRMSM | 0.36 (0.25–0.49) | 0.29 (0.17–0.43) | 47.0% (39.3%–55.6%) | 37.1% (26.9%–48.5%) | 6.2 (5.2–8.1) | 7.3 (6.2–9.1) | 0.25 (0.20–0.33) | 0.36 (0.28–0.43) |
| Test and treat (90-90)+PrEP 25% HRMSM | 0.53 (0.39–0.68) | 0.51 (0.37–0.67) | 68.3% (61.1%–75.4%) | 66.2% (57.9%–74.1%) | 4.6 (3.7–5.6) | 4.8 (3.9–5.8) | 0.12 (0.08–0.15) | 0.14 (0.10–0.18) |
| Test and treat (90-90)+PrEP 50% HRMSM | 0.56 (0.43–0.72) | 0.54 (0.40–0.70) | 72.8% (66.6%–78.7%) | 70.0% (62.2%–77.1%) | 4.1 (3.4–5.1) | 4.4 (3.6–5.3) | 0.09 (0.06–0.11) | 0.11 (0.07–0.14) |
| Test and treat (90-90)+PrEP 75% HRMSM | 0.58 (0.45–0.74) | 0.56 (0.42–0.72) | 75.2% (69.7%–80.5%) | 72.1% (64.7%–78.8%) | 3.9 (3.2–4.8) | 4.2 (3.4–5.1) | 0.07 (0.05–0.09) | 0.09 (0.06–0.12) |

(c) With PrEP effectiveness of 30%

| PrEP effectiveness=0.3 | | | | | | | | |
| --- | --- | --- | --- | --- | --- | --- | --- | --- |
| Interventions | HIV infections prevented over 20 years, million | | HIV infections prevented over 20 years, % | | HIV prevalence at 2037, % | | HIV incidence rate at 2037, per 100 person-years | |
|  | No risk compensation | With risk compensation | No risk compensation | With risk compensation | No risk compensation | With risk compensation | No risk compensation | With risk compensation |
| The baseline case | - | - | - | - | 11.2 (9.1–14.2) | 11.2 (9.1–14.2) | 0.73 (0.56–0.98) | 0.73 (0.56–0.98) |
| PrEP-25% HRMSM | 0.05 (0.03–0.09) | 0.02 (0.00–0.04) | 7.0% (4.7%–10.2%) | 2.2% (0.5%–4.8%) | 10.4 (8.6–13.2) | 10.9 (8.9–13.7) | 0.66 (0.52–0.86) | 0.70 (0.55–0.90) |
| PrEP-50% HRMSM | 0.09 (0.05–0.15) | 0.03 (0.01–0.07) | 11.7% (8.1%–16.7%) | 3.6% (0.8%–7.8%) | 9.9 (8.2–12.5) | 10.8 (8.9–13.3) | 0.62 (0.50–0.80) | 0.68 (0.54–0.87) |
| PrEP-75% HRMSM | 0.12 (0.07–0.19) | 0.04 (0.01–0.09) | 14.9% (10.4%–20.9%) | 4.6% (1.1%–9.8%) | 9.6 (8.0–12.1) | 10.7 (8.9–13.1) | 0.59 (0.48–0.76) | 0.59 (0.54–0.84) |
| Test+PrEP-25% HRMSM | 0.17 (0.10–0.28) | 0.11 (0.05–0.20) | 22.3% (16.0%–30.6%) | 13.5% (7.9%–22.0%) | 8.9 (7.7–11.0) | 9.9 (8.5–11.8) | 0.52 (0.44–0.64) | 0.60 (0.51–0.72) |
| Test+PrEP-50% HRMSM | 0.22 (0.13–0.34) | 0.11 (0.05–0.22) | 28.3% (20.9%–37.4%) | 14.6% (7.7%–24.6%) | 8.3 (7.2–10.2) | 9.8 (8.5–11.5) | 0.45 (0.40–0.57) | 0.59 (0.51–0.69) |
| Test+PrEP-75% HRMSM | 0.25 (0.15–0.38) | 0.12 (0.05–0.24) | 32.0% (24.1%–41.5%) | 15.2% (7.6%–26.3%) | 7.9 (6.8–9.7) | 9.7 (8.5–11.5) | 0.42 (0.37–0.52) | 0.58 (0.50–0.69) |
| Test and treat (90-90)+PrEP 25% HRMSM | 0.51 (0.36–0.67) | 0.48 (0.32–0.65) | 65.3% (56.6%–73.7%) | 61.2% (50.5%–71.4%) | 4.9 (4.1–5.8) | 5.4 (4.4–6.3) | 0.15 (0.11–0.19) | 0.19 (0.13–0.25) |
| Test and treat (90-90)+PrEP 50% HRMSM | 0.53 (0.38–0.69) | 0.49 (0.33–0.66) | 68.6% (60.8%–76.1%) | 62.8% (51.9%–72.9%) | 4.6 (3.8–5.4) | 5.2 (4.3–6.1) | 0.12 (0.09–0.15) | 0.17 (0.12–0.24) |
| Test and treat (90-90)+PrEP 75% HRMSM | 0.55 (0.40–0.71) | 0.50 (0.33–0.67) | 70.5% (63.2%–77.5%) | 63.8% (52.8%–73.8%) | 4.4 (3.6–5.2) | 5.1 (4.2–6.0) | 0.10 (0.08–0.13) | 0.16 (0.11–0.23) |

## Main results with time horizon of 30 years

To further test the sensitivity of the results to model assumptions, we ran a sensitivity analysis with the time horizon set to 30 years rather than 20 years. Results for this model are shown here.

### HIV epidemic projections

Without any additional intervention, HIV prevalence will become 9.7% in 2047, with peak prevalence of 12.5% in 2026, and there will be 1.06 million cumulative new infections among MSM over the next 30 years (Table S7 and Figure S4). In this scenario the HIV incidence rate will be 0.67 per 100 person-years in 2047. The sensitivity ranges of the epidemiological impact of the ten interventions on HIV prevalence and HIV incidence rates are also plotted in Figure S5.

### Impact of biomedical interventions

Test-and-treat can prevent 60.4% (0.64 million) of the total new infections estimated under the status quo scenario.

With PrEP effectiveness of 60%, the PrEP-only strategies for high-risk MSM (scenario 2 – 4) can prevent 0.13 – 0.30 million infections, 12.7 – 28.2% of the total new infections.

Compared to the base-case scenario, the combination strategies of PrEP and expanded VCT (scenario 5 – 7) could prevent 0.36 – 0.56 million new infections, 34.2 – 53.0% of the total new infections.

With a combination of test-and-treat and PrEP for 75% of high-risk MSM (scenario 10), 0.85 million new infections (i.e., 79.9% of the total new infections) could be prevented.

| (a) HIV prevalence  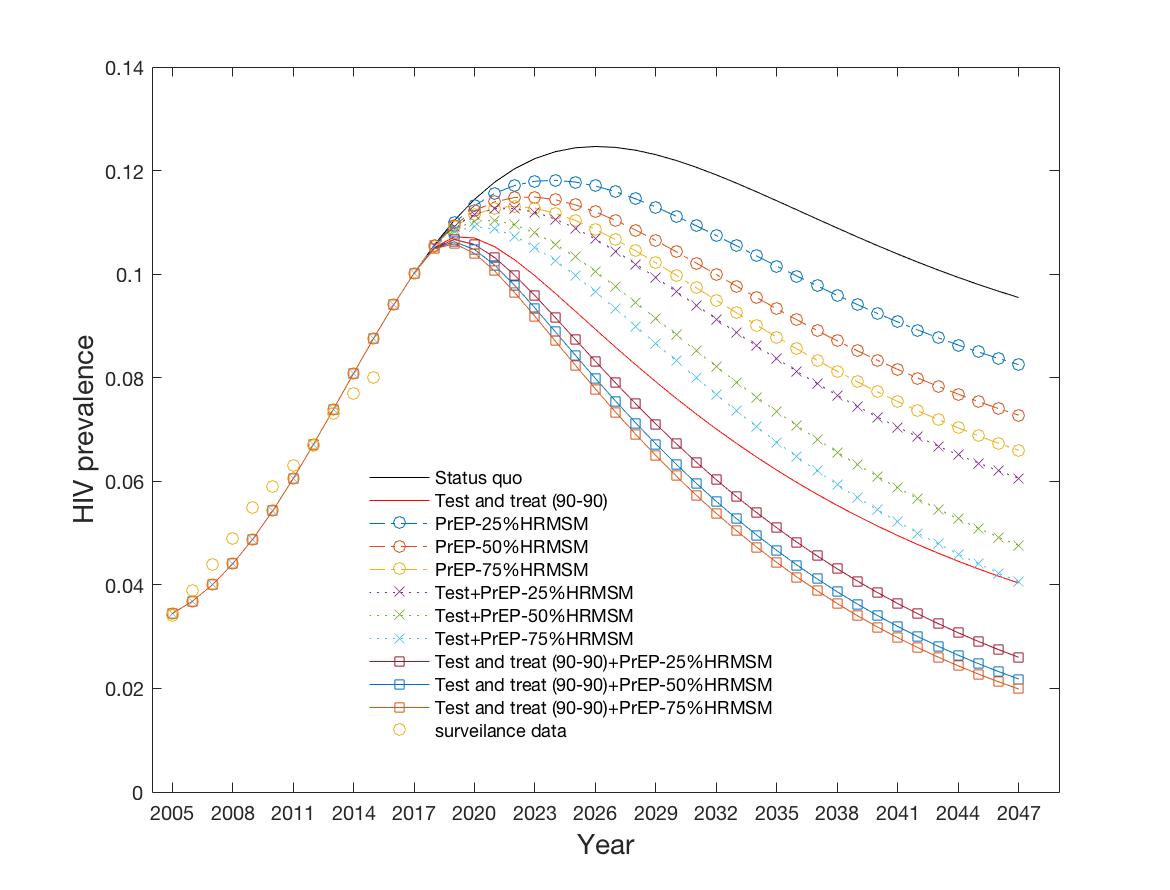 | (b) HIV incidence  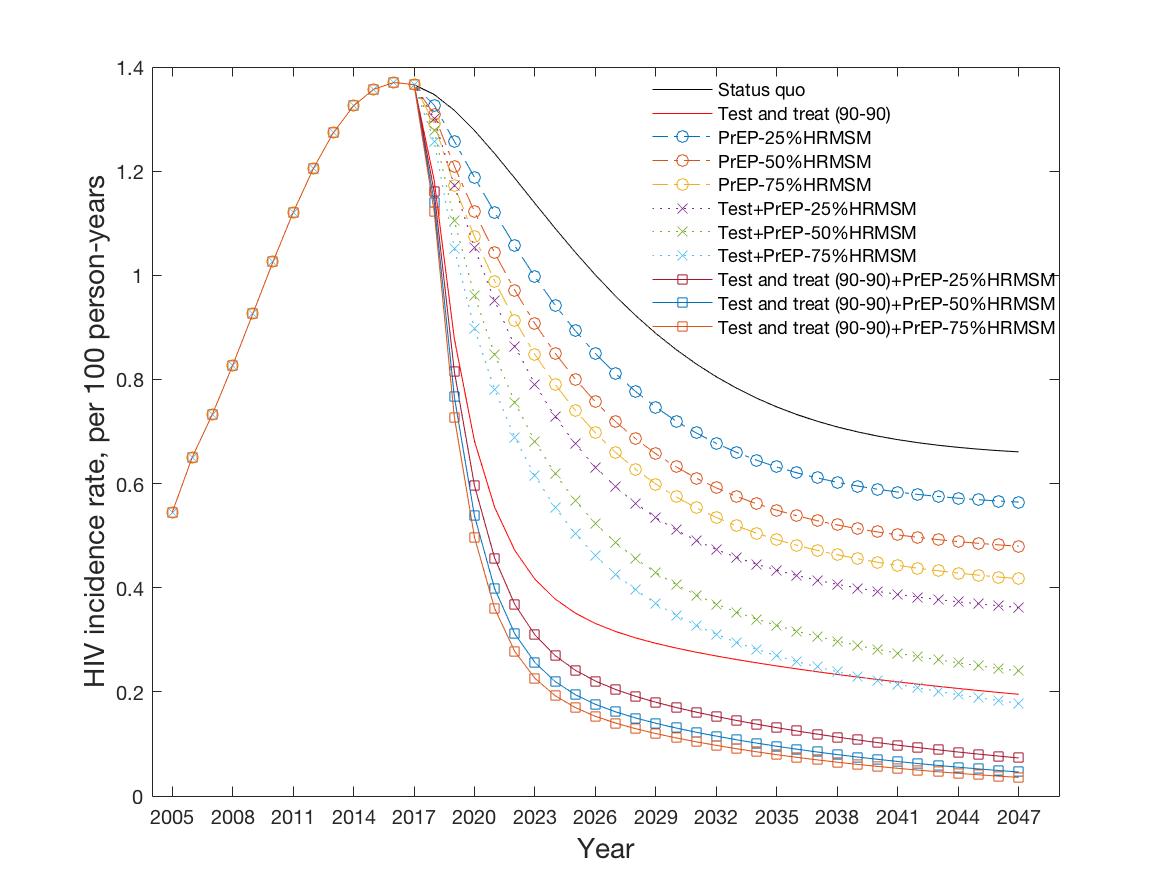 |
| --- | --- |

Figure S4 Estimated HIV prevalence and incidence between 2005 and 2047 in the status quo and the ten intervention scenarios. Left) HIV prevalence; Right) HIV incidence.

Table S7 Benefits of the individual and combined interventions, 2018~2047

|  | Total infection over 30 years (2018~2047), million | HIV infections prevented over 30 years, million | HIV infections prevented over 30 years, % | HIV prevalence at 2047, % | HIV incidence rate at 2047, per 100 person-years |
| --- | --- | --- | --- | --- | --- |
| Base case | 1.06 (0.83, 1.35) | - | - | 9.7 (7.7, 12.4) | 0.67 (0.55, 0.84) |
| Test and treat (90-90) | 0.42 (0.34, 0.51) | 0.64 (0.40, 0.91) | 60.4% (48.3%, 72.7%) | 4.0 (3.0, 4.8) | 0.19 (0.11, 0.26) |
| PrEP-25% HRMSM | 0.92 (0.76, 1.15) | 0.13 (0.07, 0.24) | 12.7% (8.8%, 19.2%) | 8.3 (7.0, 10.5) | 0.57 (0.49, 0.70) |
| PrEP-50% HRMSM | 0.83 (0.69, 1.03) | 0.23 (0.13, 0.39) | 21.8% (15.8%, 31.3%) | 7.3 (6.3, 9.3) | 0.48 (0.42, 0.60) |
| PrEP-75% HRMSM | 0.76 (0.64, 0.95) | 0.30 (0.18, 0.49) | 28.2% (21.0%, 38.8%) | 6.6 (5.7, 8.5) | 0.42 (0.36, 0.52) |
| Test+PrEP-25% HRMSM | 0.70 (0.60, 0.85) | 0.36 (0.21, 0.58) | 34.2% (25.5%, 46.0%) | 6.1 (5.3, 7.6) | 0.36 (0.29, 0.44) |
| Test+PrEP-50% HRMSM | 0.57 (0.48, 0.71) | 0.49 (0.31, 0.72) | 46.2% (37.3%, 57.6%) | 4.8 (4.0, 6.0) | 0.24 (0.17, 0.30) |
| Test+PrEP-75% HRMSM | 0.50 (0.42, 0.63) | 0.56 (0.37, 0.79) | 53.0% (44.7%, 63.4%) | 4.1 (3.3, 5.2) | 0.18 (0.12, 0.23) |
| Test and treat (90-90)+PrEP 25% HRMSM | 0.28 (0.23, 0.35) | 0.77 (0.55, 1.03) | 73.2% (65.7%, 81.0%) | 2.6 (2.0, 3.1) | 0.07 (0.04, 0.10) |
| Test and treat (90-90)+PrEP 50% HRMSM | 0.24 (0.20, 0.29) | 0.82 (0.60, 1.07) | 77.7% (71.9%, 83.8%) | 2.2 (1.7, 2.6) | 0.05 (0.03, 0.06) |
| Test and treat (90-90)+PrEP 75% HRMSM | 0.21 (0.18, 0.26) | 0.85 (0.63, 1.09) | 79.9% (75.1%, 85.3%) | 2.0 (1.6, 2.4) | 0.04 (0.02, 0.05) |

Figure S5 The effects of the ten interventions on HIV prevalence and HIV incidence rates with sensitivity range over 2005~2047, with PrEP effectiveness of 60%.

| 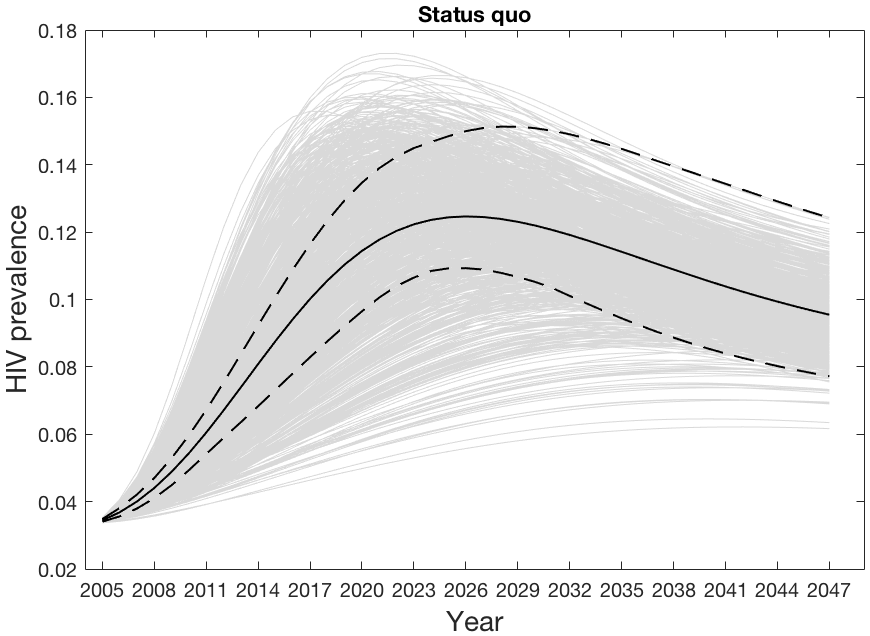 | 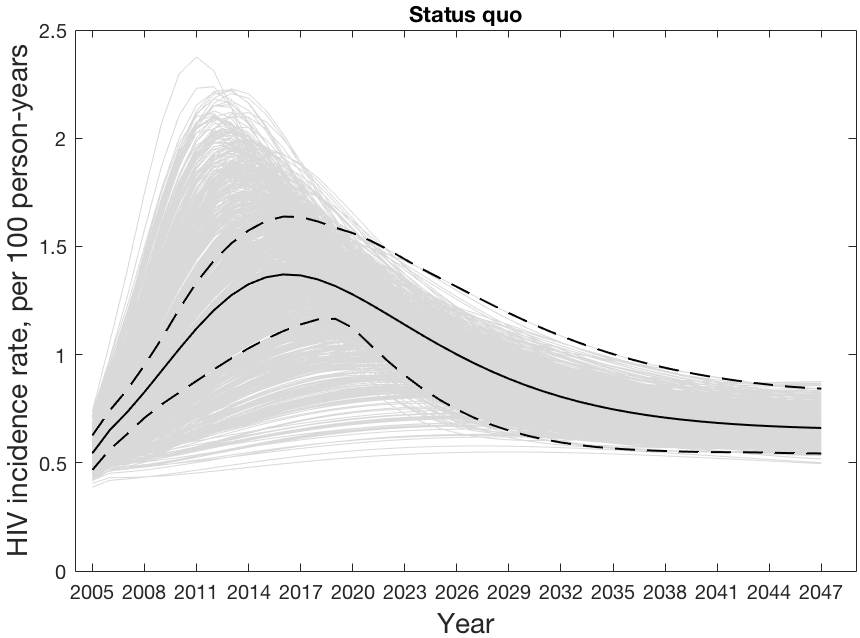 |
| --- | --- |
| 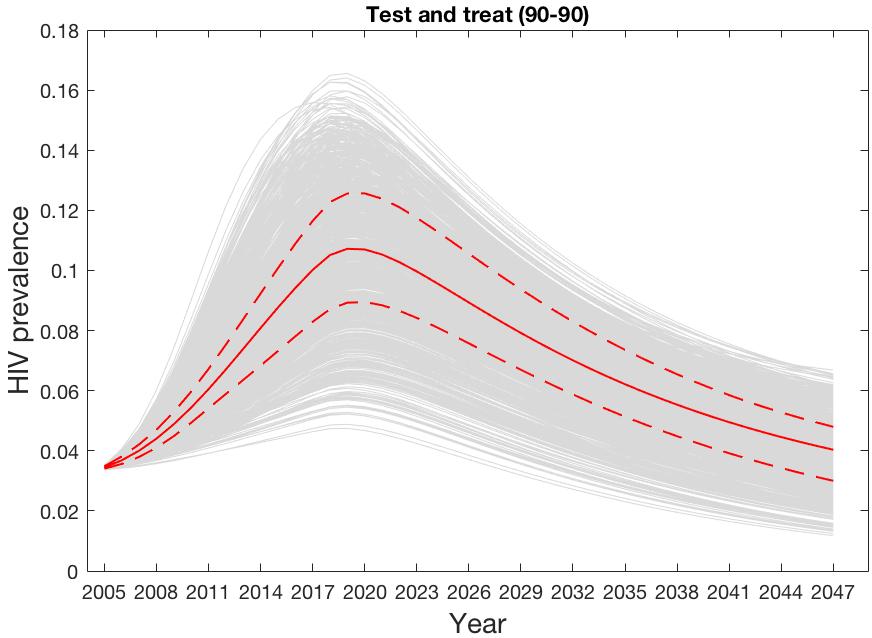 | 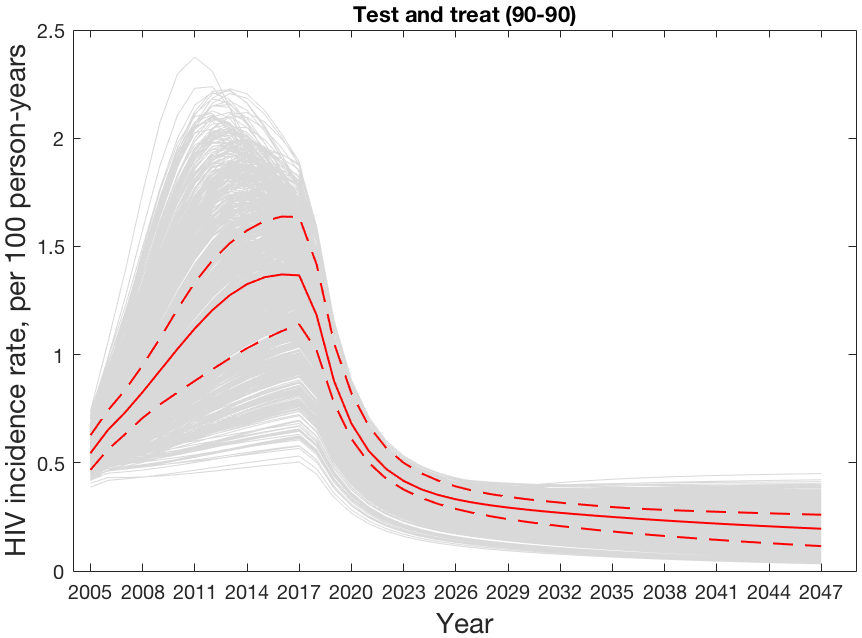 |
| 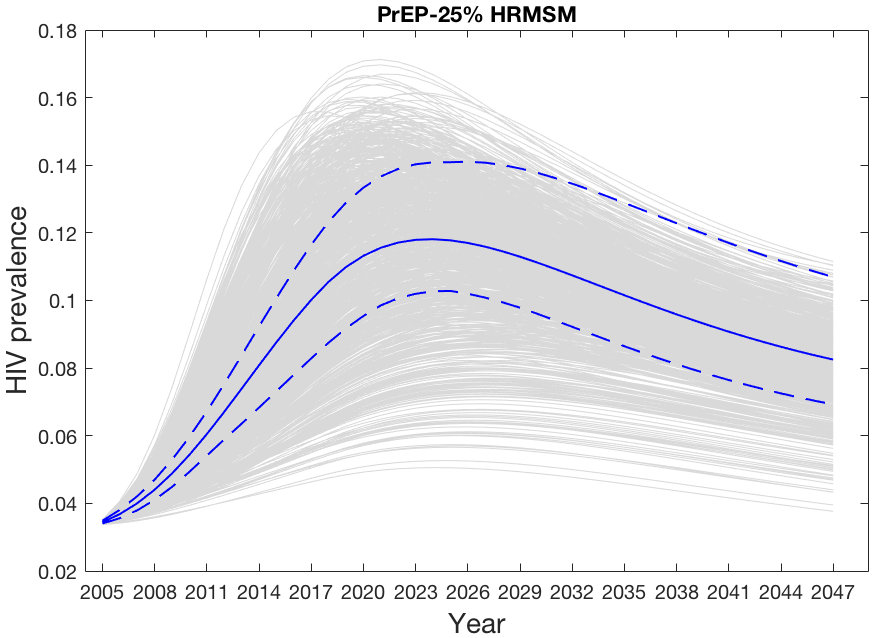 | 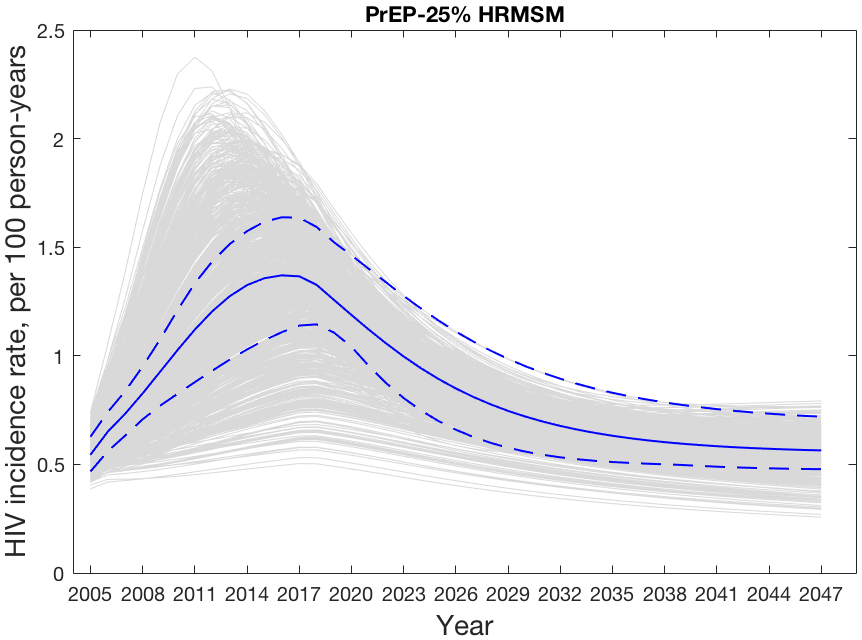 |
| 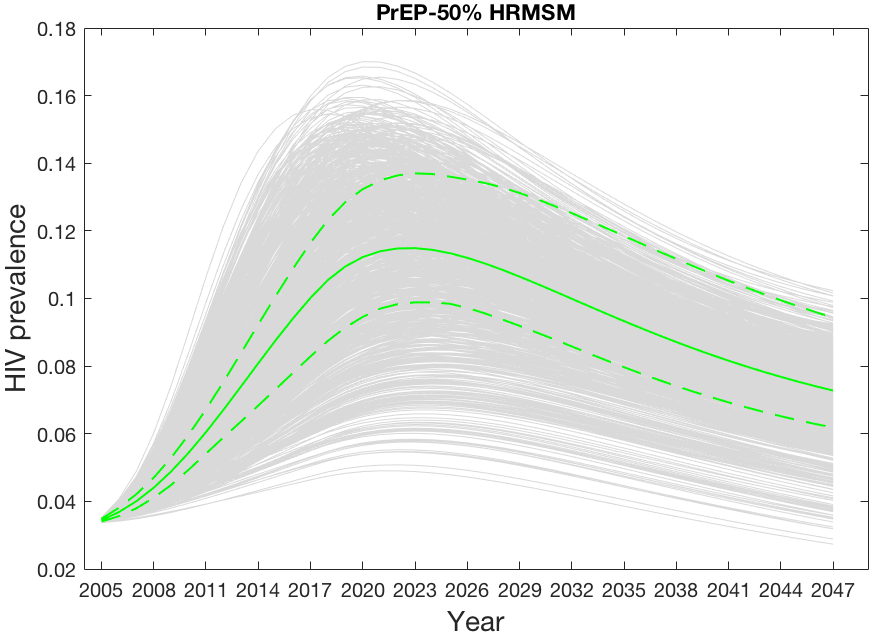 | 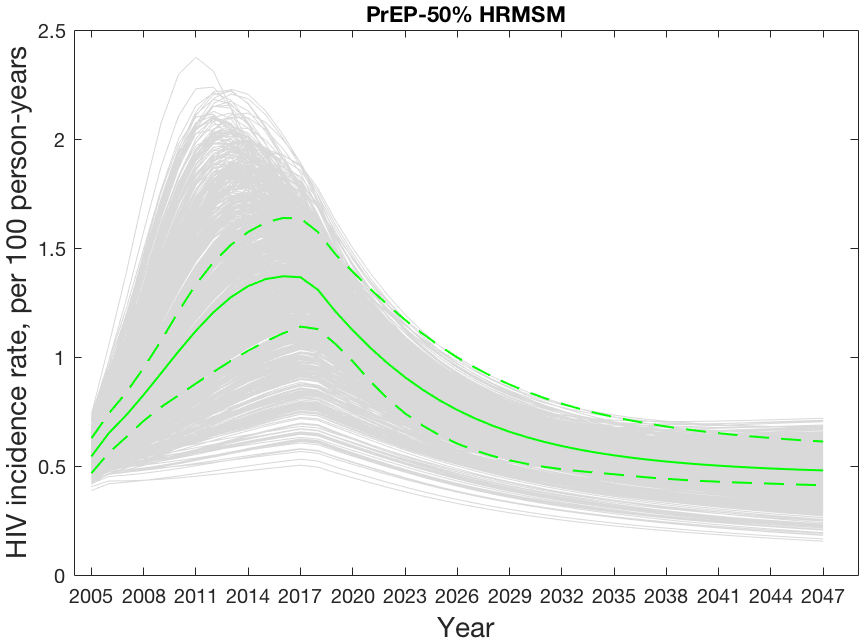 |
| 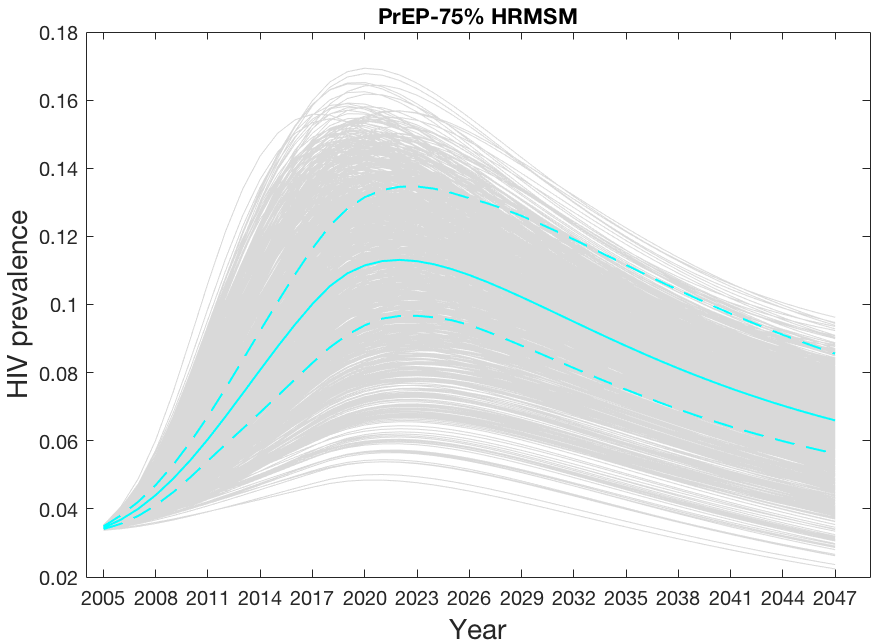 | 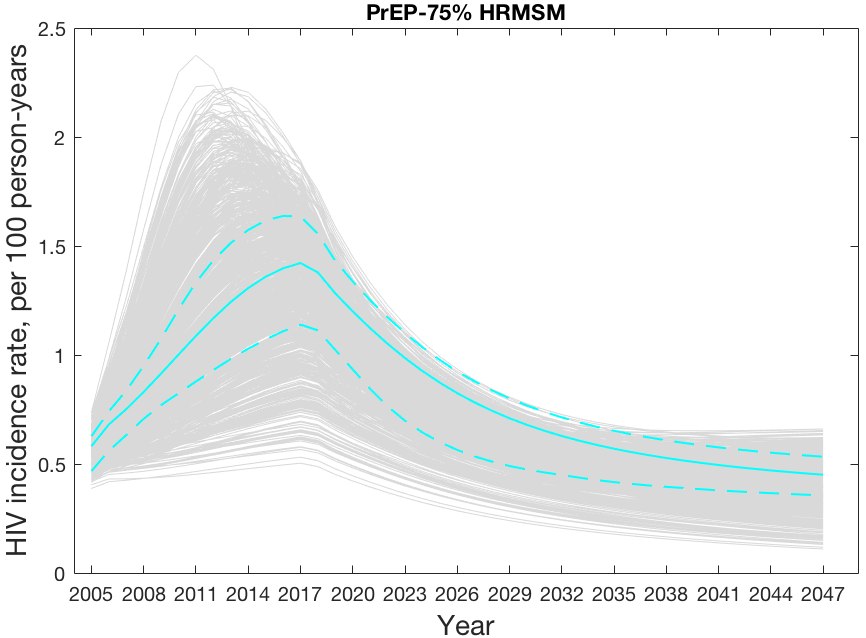 |
| 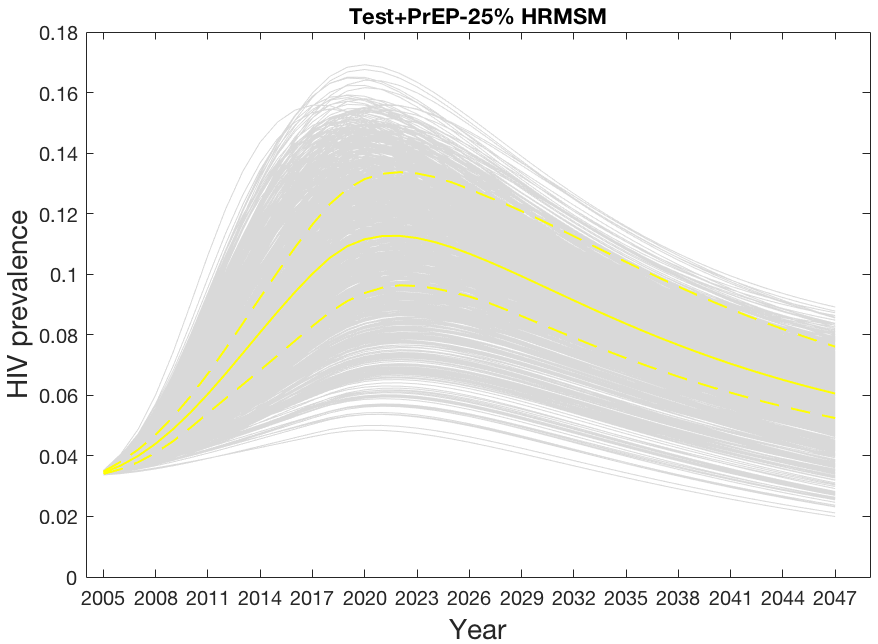 | 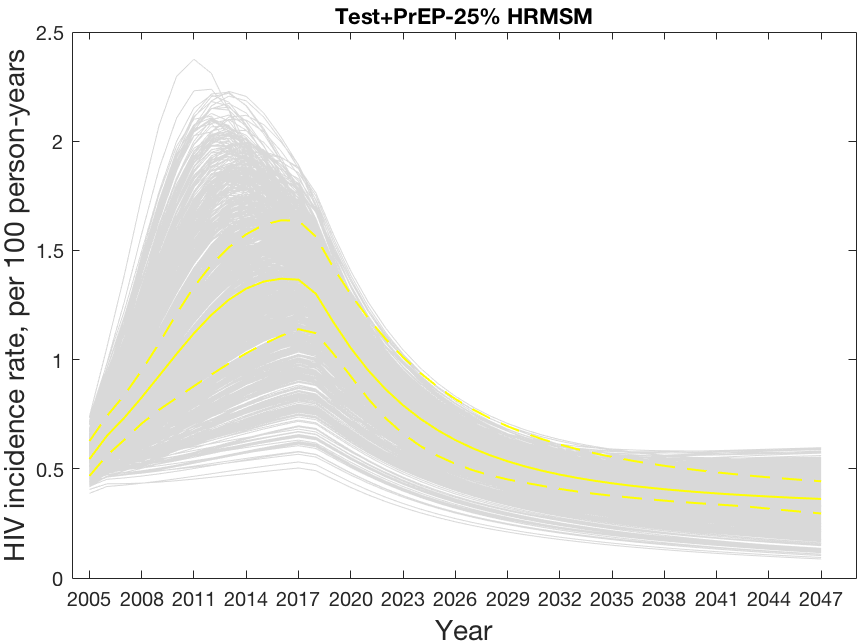 |
| 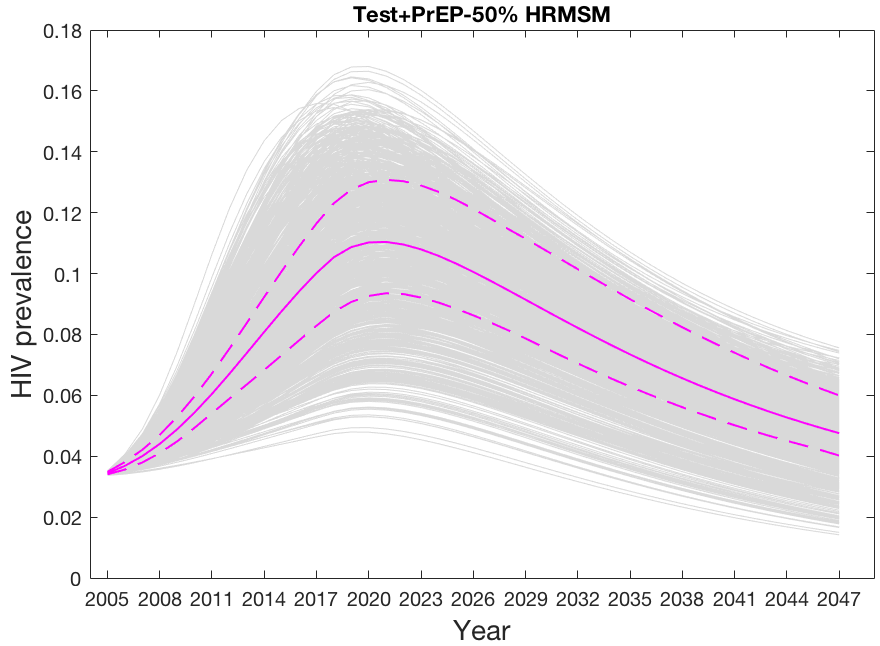 | 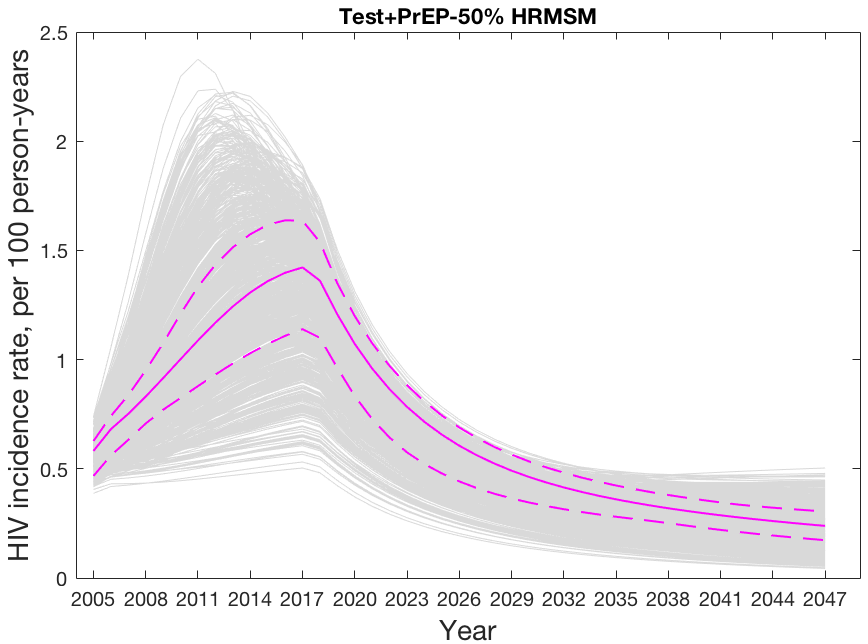 |
| 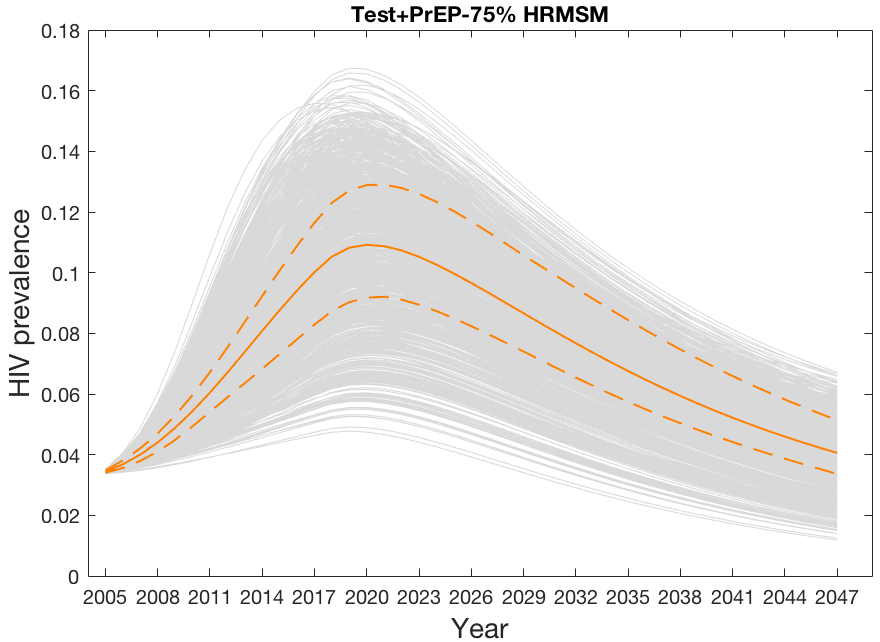 | 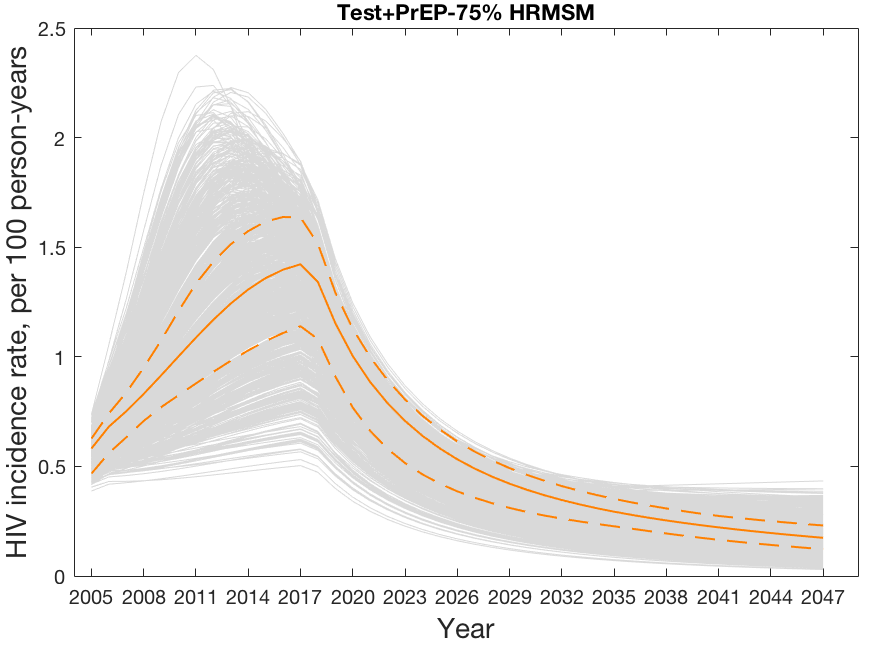 |
| 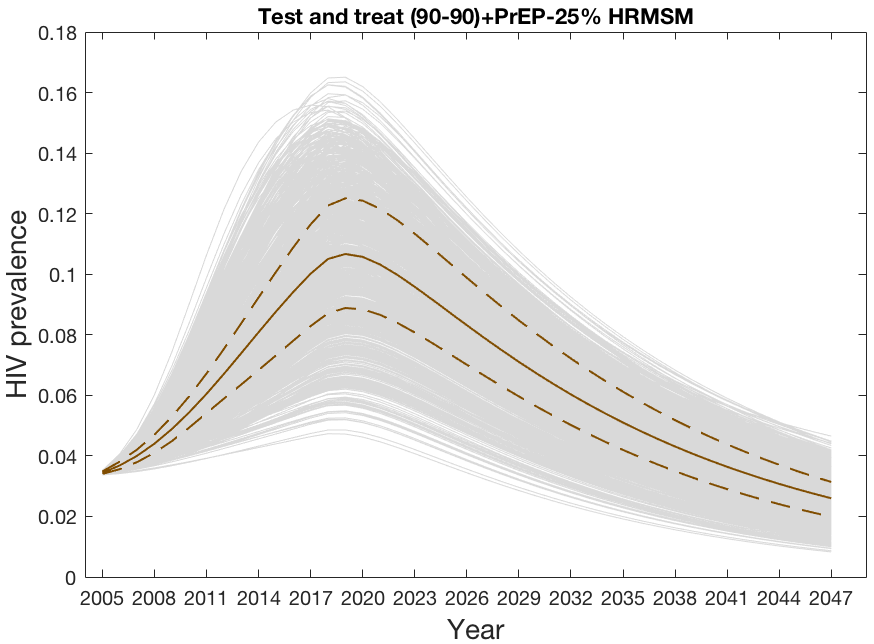 | 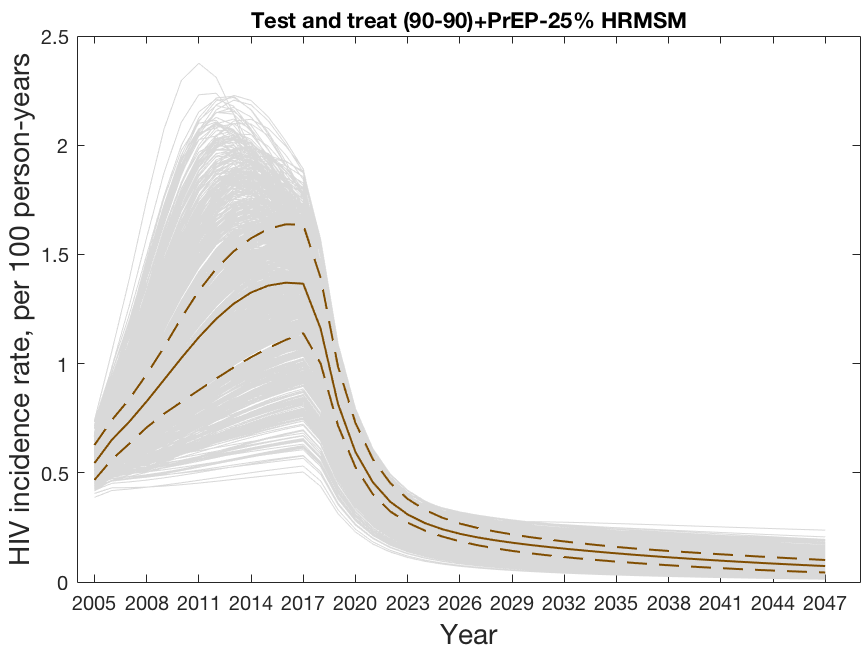 |
| 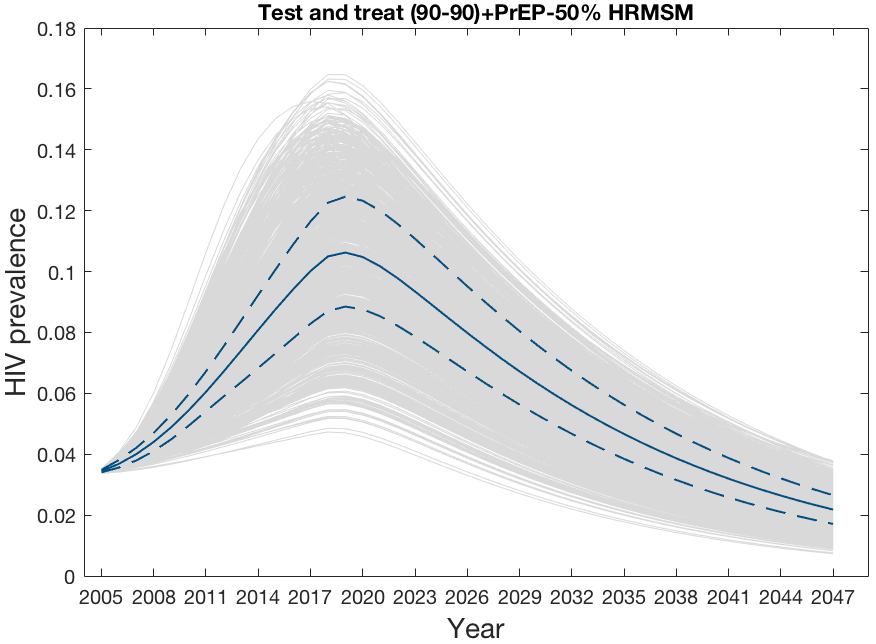 | 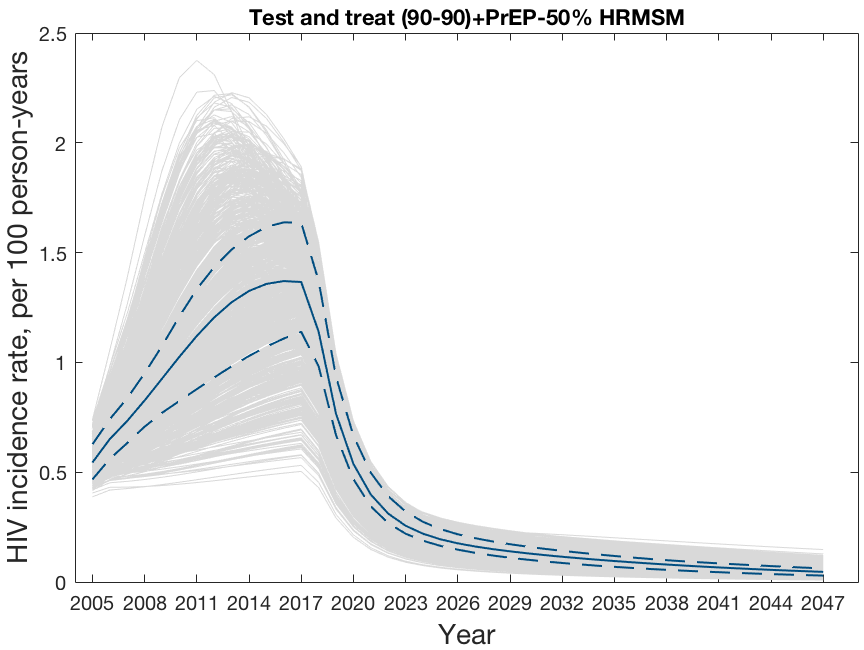 |
| 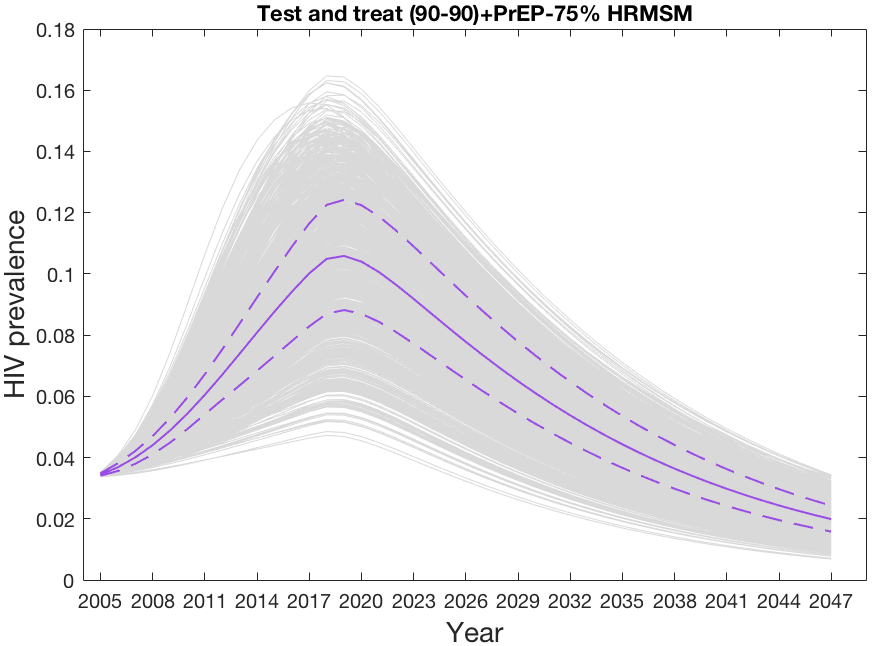 | 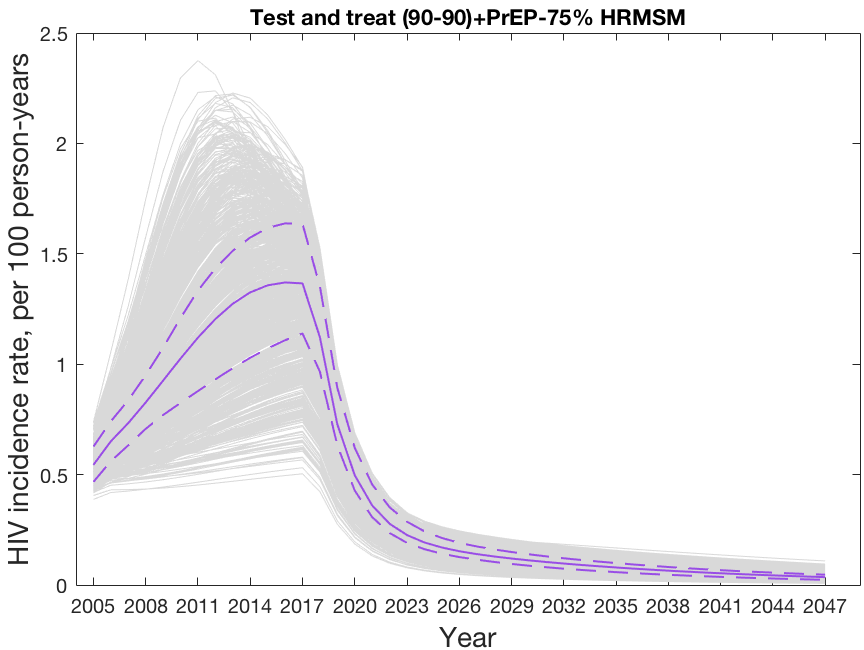 |

# References

1. National Bureau of Statistics of China. CHINA STATISTICAL YEARBOOK: China Statistics Press; 2016.

2. Palella FJ, Jr., Deloria-Knoll M, Chmiel JS, et al. Survival benefit of initiating antiretroviral therapy in HIV-infected persons in different CD4+ cell strata. *Ann Intern Med* 2003; **138**(8): 620-6.

3. Dunn D, Woodburn P, Duong T, et al. Current CD4 cell count and the short-term risk of AIDS and death before the availability of effective antiretroviral therapy in HIV-infected children and adults. *The Journal of infectious diseases* 2008; **197**(3): 398-404.

4. Ickovics JR, Hamburger ME, Vlahov D, et al. Mortality, CD4 cell count decline, and depressive symptoms among HIV-seropositive women: longitudinal analysis from the HIV Epidemiology Research Study. *JAMA* 2001; **285**(11): 1466-74.

5. Zhang F, Dou Z, Ma Y, et al. Five-year outcomes of the China National Free Antiretroviral Treatment Program. *Ann Intern Med* 2009; **151**(4): 241-51, W-52.

6. Lu F, Wang N, Wu Z, et al. Estimating the number of people at risk for and living with HIV in China in 2005: methods and results. *Sex Transm Infect* 2006; **82 Suppl 3**: iii87-91.

7. Zhang L, Chow EP, Jing J, et al. HIV prevalence in China: integration of surveillance data and a systematic review. *Lancet Infect Dis* 2013; **13**(11): 955-63.

8. Tang S, Tang W, Meyers K, Chan P, Chen Z, Tucker JD. HIV epidemiology and responses among men who have sex with men and transgender individuals in China: a scoping review. *BMC infectious diseases* 2016; **16**(1): 588.

9. MacFadden DR, Tan DH, Mishra S. Optimizing HIV pre-exposure prophylaxis implementation among men who have sex with men in a large urban centre: a dynamic modelling study. *Journal of the International AIDS Society* 2016; **19**(1): 20791.

10. Caceres CF, van Griensven GJ. Male homosexual transmission of HIV-1. *AIDS* 1994; **8**(8): 1051-61.

11. Jacquez JA, Koopman JS, Simon CP, Longini IM, Jr. Role of the primary infection in epidemics of HIV infection in gay cohorts. *J Acquir Immune Defic Syndr* 1994; **7**(11): 1169-84.

12. Mastro TD, de Vincenzi I. Probabilities of sexual HIV-1 transmission. *AIDS* 1996; **10 Suppl A**: S75-82.

13. Vittinghoff E, Douglas J, Judson F, McKirnan D, MacQueen K, Buchbinder SP. Per-contact risk of human immunodeficiency virus transmission between male sexual partners. *Am J Epidemiol* 1999; **150**(3): 306-11.

14. Zhang L, Fung Chow EP, Wilson DP. Men who have sex with men in China have relatively low numbers of sexual partners. *Infectious disease reports* 2011; **3**(1): e10.

15. Chow EP, Wilson DP, Zhang L. Patterns of Condom Use Among Men Who Have Sex with Men in China: A Systematic Review and Meta-Analysis. *AIDS and behavior* 2011.

16. Zhang B, Li X, Shi T, Cao N, Hu T. Survey on the High Risk Behaviors and Other AIDS/STI Related Factors Among Men Who Have Sex with Men (MSM) in Mainland China ('2001). *Chin J Dermatol* 2002; **35**(3): 214-6.

17. Cayley WE, Jr. Effectiveness of condoms in reducing heterosexual transmission of HIV. *Am Fam Physician* 2004; **70**(7): 1268-9.

18. Davis KR, Weller SC. The effectiveness of condoms in reducing heterosexual transmission of HIV. *Fam Plann Perspect* 1999; **31**(6): 272-9.

19. Holtgrave DR, Pinkerton SD. Updates of cost of illness and quality of life estimates for use in economic evaluations of HIV prevention programs. *J Acquir Immune Defic Syndr Hum Retrovirol* 1997; **16**(1): 54-62.

20. Ministry of Health of China. China 2010 UNGASS Country Progress Report (2008 - 2009), 2010.

21. Kamb ML, Fishbein M, Douglas JM, Jr., et al. Efficacy of risk-reduction counseling to prevent human immunodeficiency virus and sexually transmitted diseases: a randomized controlled trial. Project RESPECT Study Group. *JAMA* 1998; **280**(13): 1161-7.

22. Castilla J, Del Romero J, Hernando V, Marincovich B, Garcia S, Rodriguez C. Effectiveness of highly active antiretroviral therapy in reducing heterosexual transmission of HIV. *J Acquir Immune Defic Syndr* 2005; **40**(1): 96-101.

23. Porco TC, Martin JN, Page-Shafer KA, et al. Decline in HIV infectivity following the introduction of highly active antiretroviral therapy. *AIDS* 2004; **18**(1): 81-8.

24. Quinn TC, Wawer MJ, Sewankambo N, et al. Viral load and heterosexual transmission of human immunodeficiency virus type 1. Rakai Project Study Group. *The New England journal of medicine* 2000; **342**(13): 921-9.

25. Lodi S, Phillips A, Touloumi G, et al. Time from human immunodeficiency virus seroconversion to reaching CD4+ cell count thresholds <200, <350, and <500 Cells/mm(3): assessment of need following changes in treatment guidelines. *Clinical infectious diseases : an official publication of the Infectious Diseases Society of America* 2011; **53**(8): 817-25.

26. Sanders GD, Bayoumi AM, Sundaram V, et al. Cost-effectiveness of screening for HIV in the era of highly active antiretroviral therapy. *The New England journal of medicine* 2005; **352**(6): 570-85.

27. Honiden S, Sundaram V, Nease RF, et al. The effect of diagnosis with HIV infection on health-related quality of Life. *Quality of life research : an international journal of quality of life aspects of treatment, care and rehabilitation* 2006; **15**(1): 69-82.

28. Tsevat J, Sherman SN, McElwee JA, et al. The will to live among HIV-infected patients. *Ann Intern Med* 1999; **131**(3): 194-8.

29. Tengs TO, Lin TH. A meta-analysis of utility estimates for HIV/AIDS. *Medical decision making : an international journal of the Society for Medical Decision Making* 2002; **22**(6): 475-81.

30. Schackman BR, Goldie SJ, Freedberg KA, Losina E, Brazier J, Weinstein MC. Comparison of health state utilities using community and patient preference weights derived from a survey of patients with HIV/AIDS. *Medical decision making : an international journal of the Society for Medical Decision Making* 2002; **22**(1): 27-38.

31. Wan J, Yang G, Huang L, et al. Health Expenses and Medical Insurance among People Living with HIV/AIDS Based on Retrospective Survey in the High HIV Epidemic Area. *Medicine and Society* 2015; **28**(3): 19-22.

32. World Health Organization (WHO). Global Health Expenditure Database.

33. Zhou F, Kominski GF, Qian HZ, et al. Expenditures for the care of HIV-infected patients in rural areas in China's antiretroviral therapy programs. *BMC medicine* 2011; **9**: 6.

34. He Q, Yuan J, Xu Y, Lin P. The Projection of HIV/AIDS Medical Expenses in Guangdong province. *China J AIDS/STD* 2004; **10**(4): 271-4.

35. Cheng G, Qian Z, Hu J. Longitudinal analysis of technical efficiency of voluntary counseling and testing of HIV in China. *JOURNAL OF PEKING UNIVERSITY (HEALTH SCIENCES)* 2009; **41**(2): 135-40.

36. Long EF, Brandeau ML, Owens DK. The cost-effectiveness and population outcomes of expanded HIV screening and antiretroviral treatment in the United States. *Ann Intern Med* 2010; **153**(12): 778-89.

37. Tang H, Mao Y, Shi CX, et al. Baseline CD4 cell counts of newly diagnosed HIV cases in China: 2006-2012. *PloS one* 2014; **9**(6): e96098.

38. Vynnycky E, White RG. An introduction to infectious disease modelling. New York: Oxford University Press; 2010.
